# Nanodiamonds act as Trojan horse for intracellular delivery of metal ions to trigger cytotoxicity

**DOI:** 10.1186/s12989-014-0075-z

**Published:** 2015-02-05

**Authors:** Ying Zhu, Yu Zhang, Guosheng Shi, Jinrong Yang, Jichao Zhang, Wenxin Li, Aiguo Li, Renzhong Tai, Haiping Fang, Chunhai Fan, Qing Huang

**Affiliations:** Division of Physical Biology, and Bioimaging Center, Shanghai Synchrotron Radiation Facility, Shanghai Institute of Applied Physics, Chinese Academy of Sciences, Shanghai, 201800 China; Graduate School of the Chinese Academy of Sciences, Beijing, 100049 China; Laboratory of Water Science and Technology, Shanghai Institute of Applied Physics, Chinese Academy of Sciences, Shanghai, 201800 China; Shanghai Synchrotron Radiation Facility, Shanghai, 201203 China; School of Life Science and Technology, ShanghaiTech University, Shanghai, 200031 China

**Keywords:** Nanodiamonds, Trojan horse, Metal ion delivery, Experimental and theoretical approaches, pH-responsive ion release, Cytotoxicity

## Abstract

**Background:**

Nanomaterials hold great promise for applications in the delivery of various molecules with poor cell penetration, yet its potential for delivery of metal ions is rarely considered. Particularly, there is limited insight about the cytotoxicity triggered by nanoparticle-ion interactions. Oxidative stress is one of the major toxicological mechanisms for nanomaterials, and we propose that it may also contribute to nanoparticle-ion complexes induced cytotoxicity.

**Methods:**

To explore the potential of nanodiamonds (NDs) as vehicles for metal ion delivery, we used a broad range of experimental techniques that aimed at getting a comprehensive assessment of cell responses after exposure of NDs, metal ions, or ND-ion mixture: 3-(4,5-dimethylthiazol-2-yl)-2,5-diphenyltetrazolium bromide (MTT) assay, Trypan blue exclusion text, optical microscope observation, synchrotron-based scanning transmission X-ray microscopy (STXM) and micro X-ray fluorescence (μXRF) microscopy, inductively coupled plasma-mass spectrometry (ICP-MS), reactive oxygen species (ROS) assay and transmission electron microscopy (TEM) observation. In addition, theoretical calculation and molecular dynamics (MD) computation were used to illustrate the adsorption properties of different metal ion on NDs as well as release profile of ion from ND-ion complexes at different pH values.

**Results:**

The adsorption capacity of NDs for different metal ions was different, and the adsorption for Cu^2+^ was the most strong among divalent metal ions. These different ND-ion complexes then had different cytotoxicity by influencing the subsequent cellular responses. Detailed investigation of ND-Cu^2+^ interaction showed that the amount of released Cu^2+^ from ND-Cu^2+^ complexes at acidic lysosomal conditions was much higher than that at neutral conditions, leading to the elevation of intracellular ROS level, which triggered cytotoxicity. By theoretical approaches, we demonstrated that the functional carbon surface and cluster structures of NDs made them good vehicles for metal ions delivery.

**Conclusions:**

NDs played the Trojan horse role by allowing large amounts of metal ions accumulate into living cells followed by subsequent release of ions in the interior of cells, which then led to cytotoxicity. The present experimental and theoretical results provide useful insight into understanding of cytotoxicity triggered by nanoparticle-ion interactions, and open new ways in the interpretation of nanotoxicity.

**Electronic supplementary material:**

The online version of this article (doi:10.1186/s12989-014-0075-z) contains supplementary material, which is available to authorized users.

## Background

Since nanomaterials were discovered, a wide spectrum of them have been explored for applications in the delivery of various molecules with poor cell penetration. For example, Dai and co-workers showed that carboxylated carbon nanotubes can be conjugated with various proteins for intracellular protein delivery [1,2]. Mirkin et al. found that gold nanoparticles could introduce oligonucleotides into cells at a higher effective concentration than conventional transfection agents [3,4]. In addition, the utility of a number of nanomaterials as drug delivery platforms for water-insoluble drugs has been demonstrated [5-8]. However, few data are yet available concerning the nanoparticle-ion interactions and their bioeffects.

Nanodiamonds (NDs), a new member of nanocarbon allotropes with truncated octahedral architecture that are about 2 to 8 nm in diameter [9], integrate many of the requisite properties for various molecules delivery, including surface geometries that mediate high-affinity therapeutic binding, diversity of potential conjugates, scalability, and excellent biocompatibility [10,11]. Current researches focused on the delivery of various proteins [12,13], genes [14-16], and drugs [17-20]. In addition to that, our previous work indicates that the cellular response of NDs in serum-free medium is related to the adsorption of sodium ions by NDs [21], yet the detailed adsorption mechanism and its universal applicability for other metal ion delivery remains unclear.

**Figure 1:**
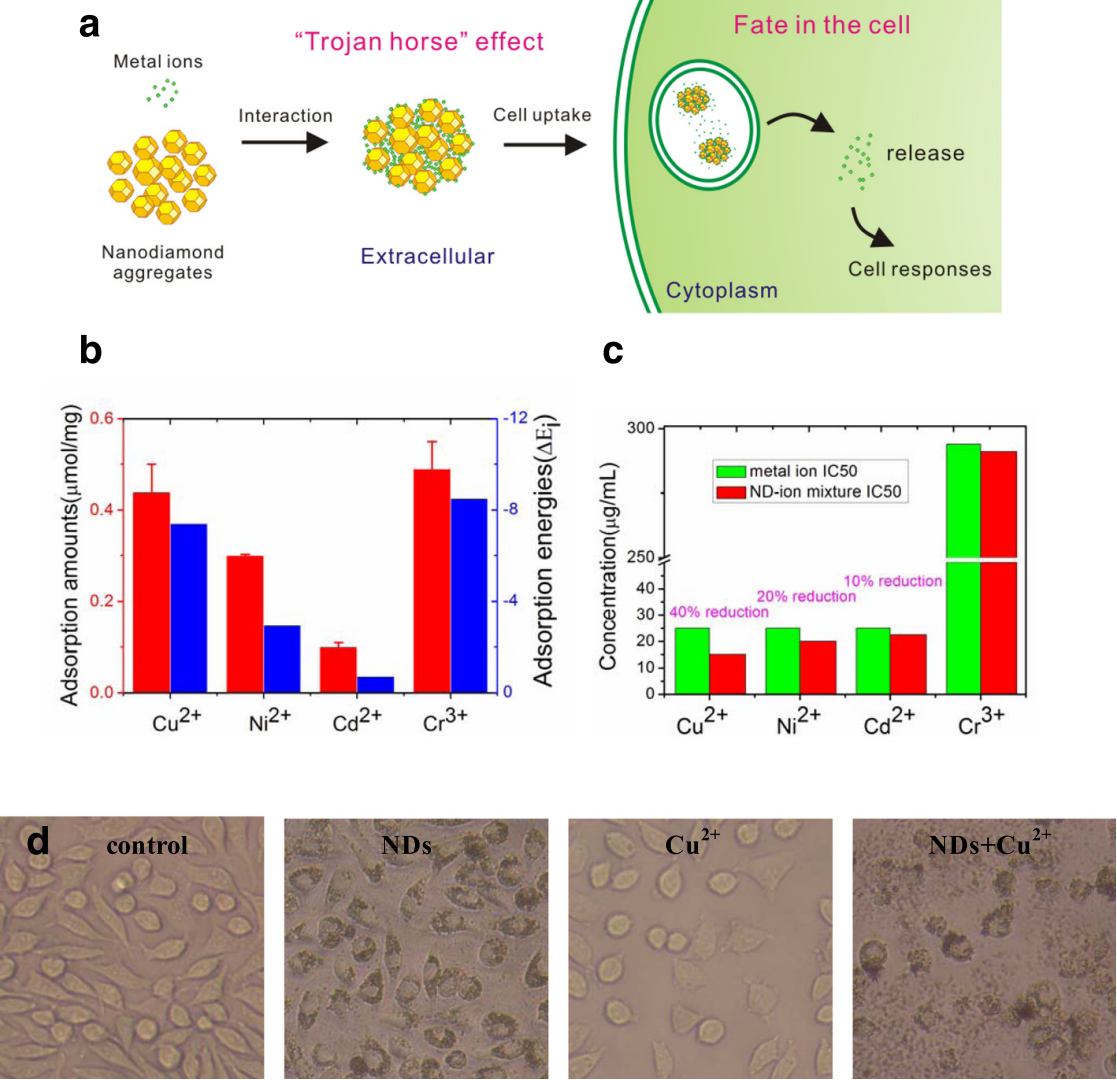
**Interactions of NDs with metal ions trigger cytotoxicity. a**: Scheme of adsorption of metal ions on NDs leads to cellular responses. **b**: The adsorption amounts (blue) and adsorption energies (red) of metal ions on NDs obtained by ICP-MS measurements and theoretical computation, respectively. **c**: The IC50 values of metal ions and ND-ion mixture and the differences between them. **d**: Optical images of L929 cells after incubation with NDs, Cu^2+^ and NDs-Cu^2+^ mixture for 24 h.

Here, we employed experimental approaches to investigate the various NDs-metal ion interactions and demonstrated that NDs acted as vehicles by allowing large amounts of metal ions accumulate into living cells followed by subsequent release of ions in the interior of cells, which then triggered cytotoxicity (Figure 1a). By theoretical approaches, the adsorption properties of different metal ion on NDs as well as release profile of ion from ND-ion complexes at different pH values were well explored. A Trojan horse type effect has been proposed to explain the biological effects of nanoparticle-biomolecule interactions [22]. The present results are also in line with a Trojan horse-type mechanism, which opens new ways in the interpretation and understanding of nanoparticle-ion interactions and their bioeffects.

## Results and discussion

### NDs-metal ions interactions trigger cytotoxicity

To study the NDs-metal ions interactions, NDs were mixed for 2 h with Cu^2+^, Ni^2+^, Cd^2+^ and Cr^3+^, which are widely dispersed in the environment and interact with living systems result in toxic effects [23,24], respectively (reached the thermodynamic equilibrium. The mean adsorption amount of Cu^2+^, Ni^2+^, Cd^2+^, Cr^3+^ for per milligram NDs determined by inductively coupled plasma-mass spectrometry (ICP-MS) is 28.1, 17.7, 12.8, and 25.6 μg, respectively (corresponding to 0.44, 0.30, 0.10 and 0.49 μmol, respectively). Density-functional theory (DFT) calculations, which are widely used in the investigation of the interactions between nanoparticles and a variety of molecules [25,26], have been used to illustrate the adsorption properties of each metal ion on NDs. An H-terminated diamond-structure nanoparticle, C_35_H_38_, one of the popular classic ND particle models [27,28], is chosen as an example. Considering the NDs used in our work are functionalized with carboxyl (-COOH) on the surface, two hydrogen atoms at the same side of the ND surface were replaced by the carboxyl (Additional file 1: Figure S1a,b). The most probable schemes of metal ions attachment to the ND surface are via deprotonated carboxyl groups (COO^—^) [21] and each ND-ion complex with the most stable structure is shown in Additional file 1: Figure S1c-h. On this basis, the single-point with a polarizable continuum model (PCM) in water is performed to calculate the adsorption energies [29,30]. Interestingly, results also show that the adsorption energies of NDs for different ions are different, and the adsorption for Cu^2+^ is the most strong among divalent metal ions, which is in striking agreement with the experimental analysis results (Figure 1b and Additional file 1: Table S1).

Our previous work indicates that large amount of sodium ions were adsorbed and delivered into the cell interior by NDs in serum-free medium, which led to obvious cell response [21]. Here, we evaluated the toxicity of ND-ion mixtures to explore whether the interaction of NDs with metal ions influenced the cellular responses. mouse fibroblast (L929) cells, which are widely used in metal ions cytotoxicity tests [31,32], were chosen as a research model. Cellular viability determination by 3-(4,5-dimethylthiazol-2-yl)-2,5-diphenyltetrazolium bromide (MTT) method shows that addition of NDs improved the metal ions induced toxicity to L929 cells with different extent (see Additional file 1: Figure S2) and the IC50 values of Cu^2+^, Ni^2+^ and Cd^2+^ decreased by approximately 40%, 20%, and 10%, respectively (Figure 1c), which has similar order to adsorption amount of each metal ion on NDs. For Cr^3+^, the adsorption amount is very similar to Cu^2+^, but the interaction of NDs with Cr^3+^ does not trigger increased cytotoxicity because cells are not sensitive to Cr^3+^ [the half maximal inhibitory concentration (IC 50) of Cr^3+^ is 294 μg/mL, excelling that of other three metal ions by ~12 times]. The effect of ND-metal ion interaction on cellular responses was again confirmed when cell viability assessments were performed for another two cells [human bronchial epithelial (BEAS-2B) cells and human keratinocyte (HaCaT) cells] exposed to metal ions and ND-ion mixtures (see Additional file 1: Figure S3).

**Figure 2:**
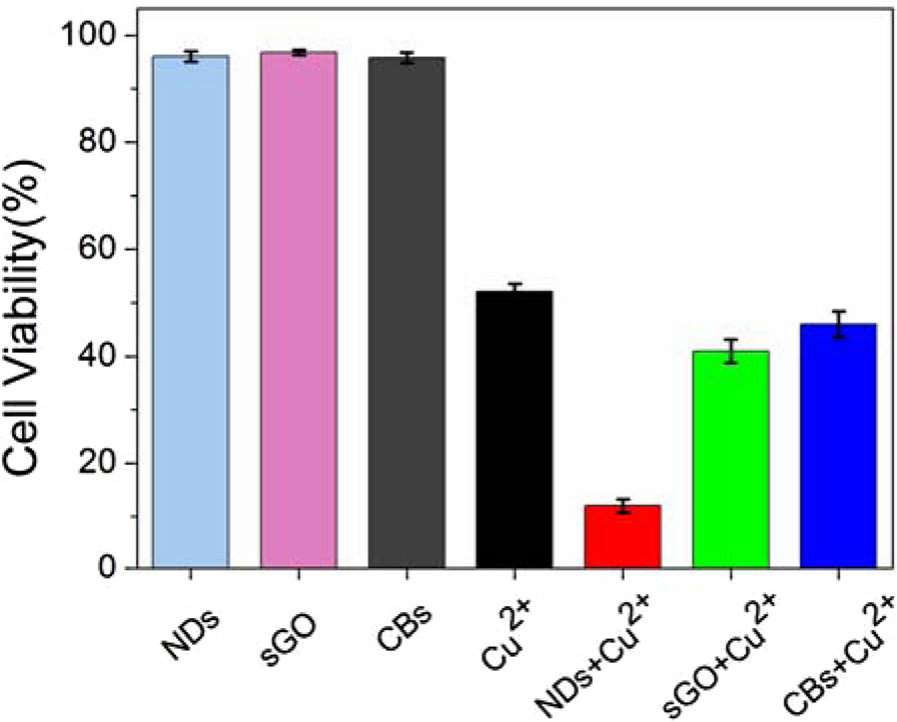
**Cu** ^**2+**^ **vectorized by NDs induced more remarkable cytotoxicity than by other nanoparticles.** Trypan blue exclusion test of cell viability after incubation with various kinds of nanoparticle-Cu^2+^ mixture for 24 h.

Since the delivery of Cu^2+^ by NDs leads to most obvious cellular responses, a detailed investigation of the interaction between NDs and Cu^2+^ is performed in the following experiment. From the kinetics curves and adsorption isotherm curves, we can see that large amounts of Cu^2+^ were adsorbed very rapidly by NDs, and saturation was achieved in less than 30 min (see Additional file 1: Figure S4). Optical microscopy (Figure 1d) images show that NDs were remarkably internalized and existed as dark granules in the cytoplasm. Addition of NDs improved the Cu^2+^ induced morphology changes and significantly reduced the cell number. Trypan blue exclusion test shows that addition of NDs decreased the cell viability from 52% to 12%, while cell survival of NDs alone was 96%. We also examined the cell viability of other nanoparticle-Cu^2+^ mixture by typan blue exclusion test. Results show that cellular responses are more remarkable when Cu^2+^ was vectorized by NDs than by ultra-small graphene oxide (sGO) or nanocarbon blacks (CBs) with similar size (Figure 2 and Additional file 1: Figure S5, S6), indicating that the unique geometries of ND clusters play an important role. NDs dispersed in aqueous solution can spontaneously form clusters with a lower free energy [33]. In our previous work [19], a model for the spatial configuration of ND clusters has been proposed. According to this model, it is speculated that metal ions can rapidly and freely diffuse along the nano-scaled pores of the ND cluster, leading to a relatively high adsorption capability.

### NDs-Cu^2+^ interactions determine their internalization fate

**Figure 3:**
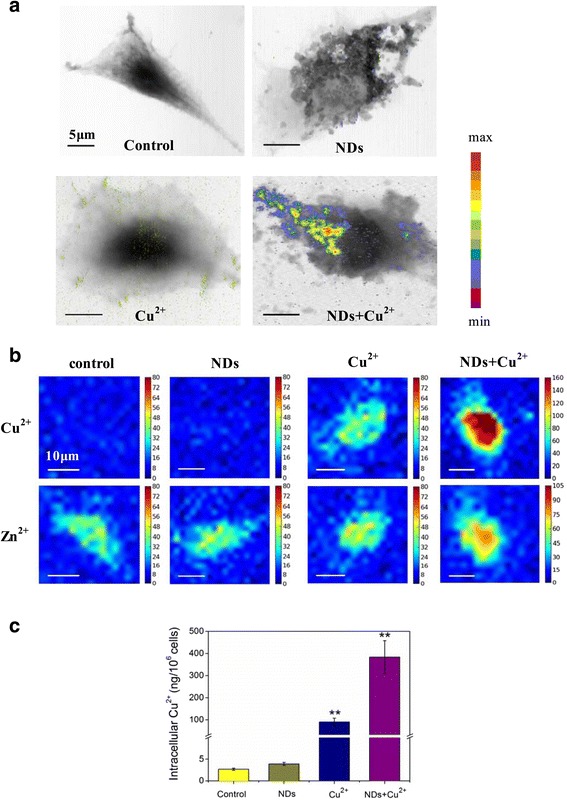
**Interactions between NDs and Cu** ^**2+**^ **determine their internalization fate. a**: STXM images of copper distribution in a typical control L929 cell (top left), cell after incubation with NDs (top right), Cu^2+^ (bottom left), and NDs-Cu^2+^ mixture (bottom right) for 24 h. The range of quantities noted by the color bar is from 3.2 × 10^−6^ to 7.0 × 10^−6^ in (top left), from 3.9 × 10^−6^ to 7.2 × 10^−6^ in (top right), from 2.4 × 10^−6^ to 7.2 × 10^−6^ in (bottom left) and from 6.5 × 10^−6^ to 5.0 × 10^−5^ in (bottom right). The scanning step was 50 nm. **b**: Imaging of intracellular copper distribution by microXRF. Elemental maps of copper (upper) and zinc (lower) are drawn. The size of a pixel is 3 μm × 3 μm. **c**: Intracellular Cu^2+^ concentration determined by ICP-MS (**p < 0.01, one-way ANOVA for comparison).

Next, we used synchrotron-based scanning transmission X-ray microscopy (STXM) and micro X-ray fluorescence (μXRF) techniques to investigate the internalization fate of free Cu^2+^ and ND-Cu^2+^ mixture. IC50 value of Cu^2+^ (25 μg/mL) calculated from Additional file 1: Figure S2 was chosen for the succedent observations. From STXM images of a typical cell, we observed a significant increase in the amount of intracellular Cu^2+^ when exposed to the ND-Cu^2+^ mixture as compared with exposure to Cu^2+^ alone. (Figure 3a and Additional file 1: Figure S7). More importantly, it is found that for the NDs-Cu^2+^ exposure groups, large amount of Cu^2+^ inside the cells were mainly attached to NDs, illustrating that Cu^2+^ entered the cells in the form of ND-Cu^2+^ complexes. Another synchrotron-based μXRF experiment was performed to further examine the difference in intracellular Cu^2+^ concentration with or without NDs. Fluorescence spectra showed that cells cultured in medium containing 25 μg/mL Cu^2+^ yielded a ≈ 18 fold increase Cu Kа signal at 8.05 KeV compared with cells cultured in basal medium (see Additional file 1: Figure S8). Consistent with recent literature [34], we found that the total amount of Zn varied little in most of the samples and the Zn concentration followed the cell shape. Additionally, uptake of NDs-Cu^2+^ complex may influence Zn ion level inside cells due to toxicity. Elemental maps of a typical cell showed that the Cu^2+^ concentration in cells treated with ND-Cu^2+^ mixture was significantly higher than that in cells treated with Cu^2+^ alone, and no Cu^2+^ signal was detected in cells treated with NDs alone, which was consistent with that treated with basal medium (Figure 3b). Further transmission electron microscopy (TEM) observation as well as energy dispersive spectroscopy (EDS) analysis confirmed these X-ray imaging results (see Additional file 1: Figure S9). Moreover, both STXM and μXRF images showed that addition of NDs made the Cu-rich zone inside cells get more concentrated.

ICP-MS was used to quantitatively measure the difference in intracellular Cu^2+^ concentration. The amount of intracellular Cu^2+^ reached 384 ± 74 ng after exposure to ND-Cu^2+^ mixture, whereas that was only 90 ± 17ng after exposure to Cu^2+^ alone (Figure 3c). All these data demonstrated that large amounts of Cu^2+^ could be delivered into cell interior through loading on NDs.

### Release profile of Cu^2+^ from ND-Cu^2+^ complex at different pH values

**Figure 4:**
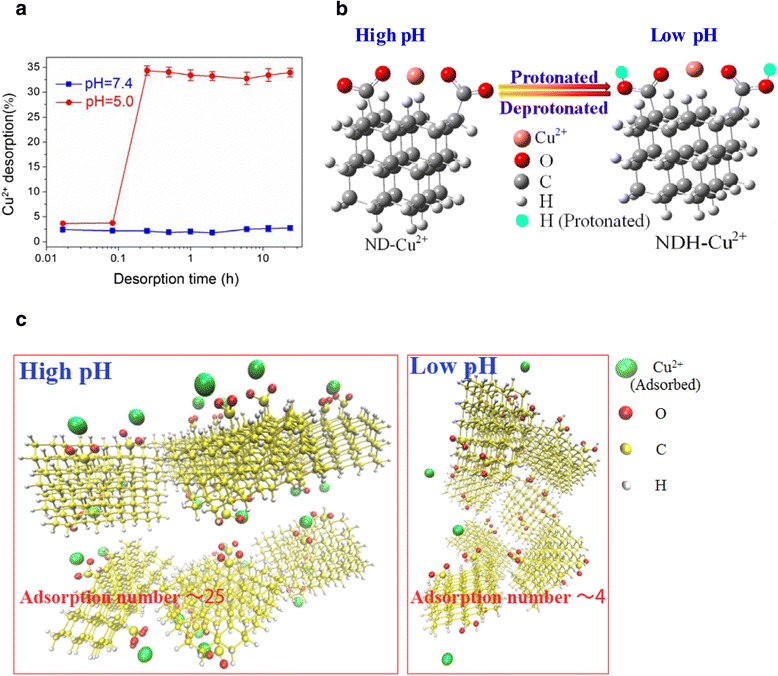
**Release profile of Cu** ^**2+**^ **from ND-Cu** ^**2+**^ **complex at different pH values. a**: Desorption amount of Cu^2+^ from ND-Cu^2+^ complexes in different pH values: pH 7.4 and pH 5.5 within 24 h. **b**: The most stable structures of the ND-Cu^2+^ complex at high and low pH (denoted by ND-Cu^2+^ and NDH-Cu^2+^, respectively) obtained by theoretical computation. **c**: Molecular modeling illustrations for the adsorption of Cu^2+^ on ND aggregates at high pH and low pH.

Several studies have shown that NDs uptake in a variety of cells was by endocytosis [10,35]. After entering the cytoplasm of the cells, many of NDs often store in endosomes, which subsequently fuse with lysosomes that contain many different hydrolytic enzymes [36]. Thus, we then examined the release profile of Cu^2+^ from ND-Cu^2+^ complex at extracellular pH (at typical cell culture medium pH of 7.4) and mildly acidic conditions (at typical lososomal pH of 5). The release curve (Figure 4a) showed that at neutral pH, a very small amount of Cu^2+^ (only about 2%) could release from ND-Cu^2+^ complex and confirmed the stability of ND-Cu^2+^ complex in cell culture medium. On the contrary, at acidic conditions, Cu^2+^ absorbed on NDs released very quickly. The amount of released Cu^2+^, presented by a weight percentage of the total Cu^2+^ adsorbed, was approximately 15 times higher than that at neutral conditions.

DFT calculations and molecular dynamics (MD) simulations are used to better understand these two different release behaviors. In the ND particle model [27,28] (details see Additional file 1: Supplementary Method), carboxyl is protonated (COOH) at low pH values and deprotonated (COO-) at high pH values (Figure 4b and Additional file 1: Figure S10) [21]. The most stable structure of ND-Cu^2+^ complex at low pH values (denoted by NDH-Cu^2+^) is shown in Additional file 1: Figure S10. PCM in water mentioned above was performed to calculate the adsorption energies of Cu^2+^ on NDs. Results indicate that it is -5.04 eV at low pH values, which is much weaker than that (-7.38 eV) at high pH values (Figure 1b and Additional file 1: Table S2). During our 4 ns MD simulations, the mean adsorption number of Cu^2+^ on NDs is 25 at high pH values and 4 at low pH values (Figure 4c) (details see Additional file 1: Supplementary Method, Additional file 2 and Additional file 3). All of these theoretical outcomes are compatible with the experimental evidences.

“Trojan horse” effect has been proposed to explain the biological effects of nanoparticle-biomolecule interactions [22]. Our results can also be explained by similar mechanism. The cellular membrane is an evolutionary developed barrier for most ions, but nanoparticles can efficiently penetrate through this membrane. Once inside the cell, Cu^2+^ releases from ND-Cu^2+^ complex, especially at low pH within the lysosomes. This pH-responsive ion release property provides new insights into interpretation and understanding of nanoparticle-ion interaction and their bioeffects.

### Enhancement of bioeffects by ND-vectorized Cu^2+^

**Figure 5:**
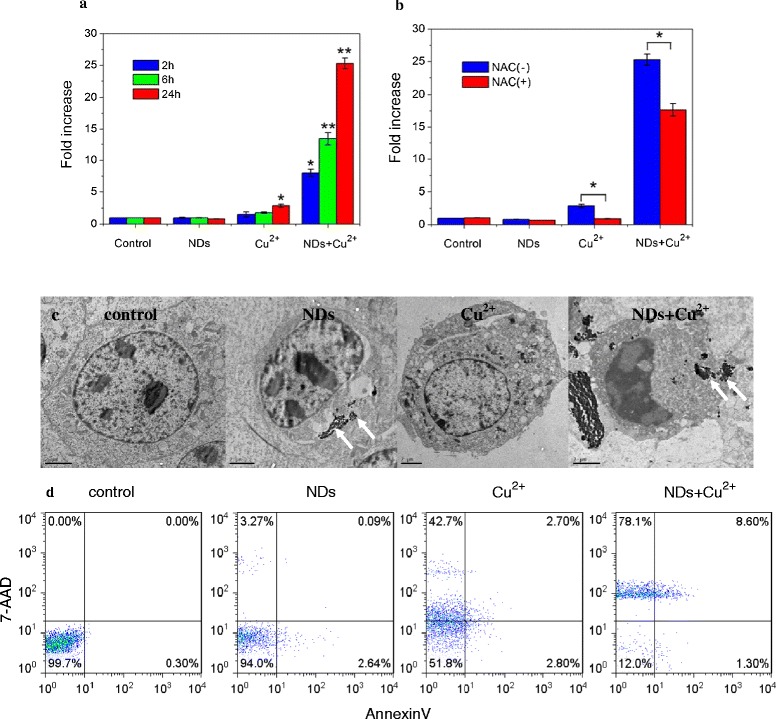
**Enhancement of bioeffects by ND-vectorized Cu** ^**2+**^ **. a** and **b**: Enhancement of Cellular ROS level by ND-vectorized Cu^2+^. **a**: ROS generation, **b**: NAC protection (*p < 0.05, **p < 0.01, one-way ANOVA for comparison). **c**: TEM images of a typical L929 cell after incubation with NDs, Cu^2+^ and NDs-Cu^2+^ mixture for 24 h. Arrows indicate NDs (Scale bars = 2 μm). **d**: Flow cytometric analysis of L929 cells after incubation with NDs, Cu^2+^ and NDs-Cu^2+^ mixture for 24 h.

Reactive oxygen species (ROS) associated oxidative stress is the general pathway for Cu^2+^ to induce bioeffects [37,38]. We then determined the intracellular ROS production to test whether this ND-Cu^2+^ interaction mediated cellular responses occurred through ROS accumulation induced by excessive Cu^2+^. Cells were loaded with the ROS measuring probe dichlorofluorescin diacetate (DCFH-DA). Results showed that after 24 h incubation, 25 μg/mL Cu^2+^ slightly increased the intracellular ROS production compared with that of control (~2.9 times) and at the other two time points (2 and 6 h), no significant increase in the intracellular ROS production was observed. However, when it is vectorized by NDs dramatically induced the production of ROS in time dependent manner, and the highest amount of ROS was generated after 24 h exposure, with ~25 fold increase over control (Figure 5a). Furthermore, pretreatment with *N*-Acetylcysteine (NAC, ROS scavenger [39]) for 2 h provided effective protection against Cu^2+^ induced ROS generation (Figure 5b).

Increased ROS generation has been suggested to associate well with cytotoxicity. TEM studies demonstrated that large amounts of NDs were internalized into cells and ND-vectorized Cu^2+^ lead to severe cell apoptosis and necrosis (Figure 5c). Quantitative flow cytometric analysis further showed that compared with Cu^2+^ exposure alone, the addition of NDs significantly enhanced the percentage of cells stained positive for 7-Amino-actinomycin (7-AAD), indicating a loss of membrane integrity which is clearly suggestive of necrotic cell death (Figure 5d). However, after exposure to NDs alone, no significant increase in the percentage of cells stained positive for 7-AAD or Annexin V when compared to control. Taken together, our data indicated that large amounts of Cu^2+^ delivered into cells by NDs led to the elevation of intracellular ROS level, which triggered cytotoxicity.

Previous studies have indicated that in the synthesis of nanomaterials such as metal-containing fullerenes and carbon nanotubes, many metal impurities were also bioavailable at toxicologically significant concentrations despite metal bioavailability varied greatly from sample to sample [40,41]. In this work, by using both experimental and theoretical approaches, we demonstrated the potential of NDs as vehicles for intracellular delivery of several kinds of metal ions. More importantly, detailed investigations of the interaction between NDs and Cu^2+^ as well as their bioeffects indicated that the released Cu^2+^ from ND-Cu^2+^ complexes in the low-pH-intracellular environment led to the elevation of intracellular ROS level, which triggered cytotoxicity. All of these results suggest that we should pay more attention to the relative contributions of metal ions internalized by cells, through a Trojan horse mechanism, on overall cytotoxicity.

## Conclusions

In conclusion, we have demonstrated the interactions between NDs and metal ions, Cu^2+^, Ni^2+^, Cd^2+^, and Cr^3+^, using both experimental and theoretical approaches. Results showed that adsorption capacity of NDs for different metal ions was different, and among them, the adsorption for Cu^2+^ was the most strong. These different ND-ion complexes had different cytotoxicity by influencing the subsequent cellular responses. Detailed investigation of cell responses of ND-Cu^2+^ complexes demonstrated that NDs played the Trojan horse role by allowing more Cu^2+^ accumulate into living cells followed by subsequent release of Cu^2+^ in the low-pH-intracellular environment leading to higher ROS level. These results provide therefore the evidence of the cytotoxicity triggered by nanoparticle-ion interactions, and surely open new ways in the interpretation and understanding of nanotoxicity.

## Methods

### Materials

NDs with individual sizes of 2–10 nm synthesized by detonation techniques were supplied by Gansu Gold Stone Nano. Material. Co. Ltd., China. They were easily dispersed in aqueous solution by sonication. Resultant suspensions were stable for extended periods of time. TEM images showed that the size of the majority of ND clusters was about 40-200 nm. The details for characterization have been described in our previous work [19].

Metal chlorides including CuCl_2_ · 2H_2_O, NiCl_2_ · 6H_2_O, CdCl_2_ · 2.5H_2_O, and CrCl_3_ · 6H_2_O were obtained from Sigma-Aldrich, China. Stock solution of each metal ion was prepared with distilled millipore water and was used to make serial dilutions. All other chemicals used were of analytical grade.

### Determination of adsorption amount of each metal ion on NDs

Each metal ion solution (250 μg/mL) was mixed thoroughly with the aqueous ND solution (1mg/mL) for 2 h at 37°C. After centrifugation at 13,000 rpm for 20 min, the concentration of metal ions in the supernatant was determined by inductively coupled plasma-mass spectrometry (ICP-MS, X-7, Thermo Elemental, USA). The amounts of metal ions adsorbed on the NDs were obtained by subtraction.

### Theoretical computational methods

The B3LYP [42] method within generalized gradient approximation in the framework of DFT is employed to examine the intermolecular interactions between the ND particle and metal ions (Cu^2+^, Ni^2+^, Cd^2+^ and Cr^3+^). The electron wave functions in the Gaussian function basis are used. We performed the optimized stationary points via the Berny algorithm [43]. For geometry optimization, we employed the double-ζ basis and added a diffuse function to the basis set, using a d-polarization function to carbon and oxygen atoms as well as one p-polarization function to the hydrogen atoms (marked with 6-31 + G(d,p)). At the same time, the pseudopotential function with Lanl2dz was introduced into the basis set for each metal ion. Furthermore, the single-points at this method with a PCM [30] in water were performed to show the solution effects. The adsorption energy of each metal ion on a single ND or NDH particle (**ΔE**_i_) was defined as:

$$ \boldsymbol{\Delta} {\mathbf{E}}_{\mathbf{i}}={\mathbf{E}}_{\mathbf{ND}\mathbf{\hbox{-}}\mathbf{i}}\mathbf{\hbox{-}}{\mathbf{E}}_{\mathbf{ND}}\mathbf{\hbox{-}}{\mathbf{E}}_{\mathbf{i}} $$

where **E**_ND-i_, **E**_ND_, and **E**_i_ are total energy of metal ions adsorbed on a single ND particle, a single ND particle and metal ions, respectively. All calculations were carried out using the Gaussian-09 package [44].

### Cytotoxicity assessment of metal ions and metal-ion mixtures

L929 cells were grown in RPMI-1640 (Gibco) cell culture medium supplemented with 10% fetal bovine serum (FBS), and the resultant cell suspension (7 × 10^4^ cells/mL) was dispensed into 24-well plates and incubated overnight to allow for cell adherence. After washing twice with phosphate buffered saline (PBS), cells were treated with Cu^2+^ (0 to 40 μg/mL), Ni^2+^ (0 to 40 μg/mL), Cd^2+^ (0 to 30 μg/mL), Cr^3+^ (0 to 400 μg/mL) with or without 50 μg/mL NDs for 24 h. Cells incubated with the complete culture medium were used as controls. For ND-metal ion mixture samples, each kind of metal ion solution was mixed thoroughly with the aqueous ND solution for 2 h prior to experiments. Cell viability was determined by the MTT (Sigma-Aldrich, Shanghai, China) assay. The cell viabilities were expressed as a percentage of OD_test_/OD_control_. All of the viability assessment data was based on three independent measurements. The IC 50 value of each metal ion and NDs-ion mixture was determined by Origin Pro 7.5 software.

BEAS-2B cells were grown in RPMI-1640 cell culture medium supplemented with 10% FBS. HaCaT cells were grown in DMEM (Gibco) cell culture medium supplemented with 15% FBS. Cells were exposed to 50 μg/mL NDs, 25 μg/mL Cu^2+^, 25 μg/mL Ni^2+^ (IC50 value of each metal ion for L929 cells), NDs-Cu^2+^ mixture and NDs-Ni^2+^ mixture for 24 h, respectively. The toxicity of these kinds of metal ions and metal-ion mixtures to BEAS-2B and HaCaT cells was determined using the same method as described above. All of these cell lines were selected based on their common use in metal ions cytotoxicity tests. In addition, they are nontumorogenic [31,32,45,46].

### Cytotoxicity assessment of Cu^2+^ and NDs-Cu^2+^ mixture

A L929 cell suspension (7 × 10^4^ cells/mL) was dispensed into 24-well plates and incubated overnight to allow for cell adherence. After washing twice with PBS, 50 μg/mL NDs, 25 μg/mL Cu^2+^ (IC50 value) and NDs-Cu^2+^ mixture were added into the plate wells. Cells incubated with the complete culture medium were used as controls. Following 24 h incubation, the cell morphology changes were observed and recorded using an Olympus CKX41SF inverted optical microscope at 200 × magnification. Cell aliquots were collected and immediately stained with trypan blue for 5 min. Cell proliferation was measured by counting cell numbers and cell viabilities were expressed as a percentage of Number_test_/Number_control_.

In flow cytometric assays, cells were seeded in 6-well plates. After exposure to NDs, Cu^2+^ and NDs-Cu^2+^ mixture for 24 h, cells were stained as described in the methods for using the BD FACSArrayTM Bioanlysis (BD Biosciences). Apoptotic cells were quantitated with APC-labeled Annexin V and the vital dye 7-AAD.

### Observation of Cu^2+^ delivery into the cells by NDs

To observe the NDs as vehicles for intracellular Cu^2+^ delivery by STXM techniques, the Si_3_N_4_ windows (100 nm thickness) were previously put into 24-well plates and sterilized by ultraviolet rays. A L929 cell suspension (7 × 10^4^ cells/mL) was dispensed into 24-well plates and incubated overnight to allow for cell adherence to the Si_3_N_4_ windows. NDs, Cu^2+^ and NDs-Cu^2+^ mixture were added into the designated wells by the same method as described above, and the cells were incubated for 24 h and then fixed with a few of 4% paraformaldehyde in 0.1M PBS. After wash with PBS, the cells were then dehydrated in a graded gradient ethanol series and dried under air. The ratio-contrast imaging of dual-energy absorption for copper mapping was performed at the beamline BL08U1 of Shanghai Synchrotron Radiation Facility (SSRF). A Fresnel zone plate focuses mono-energetic X-rays provided by SX700 monochromator and the focal beam point is 30 nm in diameter. Two photon energies were chosen, E1 = 936.6 eV and E2 = 934 eV, which are just above and below the absorption edge of copper, to scan the sample pixel by pixel. A K-edge division method [47] was applied to obtain the overlay of absorption-contrast images of cells and copper distribution images.

Another synchrotron-based μXRF microscopy was used to further verify the difference in intracellular Cu^2+^ concentration with or without NDs. The Mylar X-ray films (Hoffman, 12 μm thickness) were previously put into 24-well plates and sterilized by successive baths in 70% ethanol. A L929 cell suspension (7 × 10^4^ cells/mL) was dispensed into 24-well plates and incubated overnight to allow for cell adherence to the thin films. NDs, Cu^2+^ and NDs-Cu^2+^ mixture were added to the designated wells by the same method as described above. Following incubation, cells were washed with PBS and fixed with ice cold 80% ethanol. After excess ethanol had evaporated, the μXRF microscopy was performed at the beamline BL15U1 of SSRF. Incident x-rays energy of 10 keV, obtained with a Si (111) monochromator, was chosen in order to excite the K-lines of X-ray fluorescence of elements from P to Zn. A light microscope was coupled to a computer for sample viewing and the sample platform was moved by a motorized X-ray mapping stage. A Kirkpatrick-Baez mirror system focused the x-ray beam to a spot size of 3μm × 3μm on the specimen, which was raster-scanned. XRF from the specimen was captured with an energy dispersive silicon drift detector (Vortex, USA). From the analysis of the X-ray fluorescence spectrum for each pixel, a spatial image can be obtained for each element separately. Such an image represents a two-dimensional projection of the volumetric distribution of the elements. The vertical and horizontal pixel size was 2μm each. Data collection time for each pixel was 4s and fitting of the fluorescence data has been performed in batch processing using the PyMca 4.0.9 software [48]. Three cells in each sample were scanned and images of a typical cell were shown.

### Determination the intracellular Cu^2+^ concentration

Cells were treated with NDs, Cu^2+^ and NDs-Cu^2+^ mixture by the same method as mentioned above. Cells without any treatment were used as controls. After incubation, all samples were washed thoroughly with PBS for three times and cells were trypsinized, collected and transferred to Enppendorf tubes. Aliquots of 100 μl were taken from the each sample to determine the number of cells, and the remainder of the cell suspensions was sonicated for 3-5 min. The cell supernatant after sonication was digested for 12 h by 2% nitric acid and then analyzed the Cu^2+^ concentration by ICP-MS.

### Desorption of Cu^2+^ from NDs-Cu^2+^ complexes

NDs-Cu^2+^ complexes were prepared by the same method as described in “adsorption of metal ions on NDs” section. After gentle wash with millipore water, the NDs-Cu^2+^ complexes were resuspended in PBS solution at pH 7.4 and pH 5.0, respectively. Following sonication, the mixture was put aside over the course of 24 h. At a given interval time, mixtures were centrifuged at 13,000 rpm for 20 min and the concentration of Cu^2+^ in the supernatant was determined by ICP-MS. The variation of desorption amounts with the elapsed times gave release curve of Cu^2+^ from NDs-Cu^2+^ complex.

### Determination of intracellular reactive oxygen species level

Cells were treated with NDs, Cu^2+^ and NDs-Cu^2+^ mixture as mentioned above. Cells without any treatment were used as controls. After 2, 6, and 24 h incubation, cells were washed with PBS and loaded with DCFH-DA (5 μM) from Reactive Oxygen Species Assay Kit (Beyotime Institute of Biotechnology, Jiangsu, China) for 40 min. After a further wash with PBS, cell suspensions were collected into 96-well flat bottom black plate to determine the relative fluorescent intensity (RFI, λ_ex_ 485 nm, λ_em_ 535 nm) by Tecan GENios fluorescence microplate reader. The RPMI-1640 medium was used as blank. The RFI over control was calculated as the measured ROS levels.

In order to observe the protection effect of NAC, an antioxidant agent, against oxidative stress induced by Cu^2+^, cells were pretreated with NAC (Sigma-Aldrich, China, 10 mM) for 2 h. After wash with PBS for three times, cells were treated with NDs, Cu^2+^ and NDs-Cu^2+^ mixture for 24 h. Cells without any treatment were used as controls. The subsequent DCFH-DA loading and intracellular RFI determination were conducted using the methods mentioned above.

### TEM observation

After exposing L929 cells to NDs, Cu^2+^ and ND-Cu^2+^ mixture for 24 h, the cells were washed twice with PBS, and prefixed with a few of 2.5% glutaraldehyde in 0.1 M PBS. Then, the cells were collected using a cell scraper and centrifuged at 2000 rpm for 10 min. Cell aggregates were fixed in 2.5% glutaraldehyde for at least 2 h. After a further wash with PBS, the cells were then dehydrated in a graded gradient ethanol series and embedded in Epon618. Ultrathin sections of the embedded cells were then examined by TEM.

### Statistical analysis

All results are expressed as the mean ± standard deviation from triplicate experiments performed in a parallel manner unless otherwise indicated. Statistical significance of the data was determined by *t*-tests or one-way analysis of variance (ANOVA) using SPSS 11.5. ** equals P < 0.01; * equals P < 0.05.

## Additional files

Additional file 1:
**Supplementary information.**
Additional file 2:
**Movie of Cu2+ adsorption on the ND particles.**
Additional file 3:
**Movie of Cu2+ adsorption on the NDH particles.**

## Abbreviations

NDs: Nanodiamonds
L929: Mouse fibroblast cells
MTT: 3-(4,5-dimethylthiazol-2-yl)-2,5-diphenyltetrazolium bromide
BEAS-2B: Human bronchial epithelial cells
HaCaT: Human keratinocyte cells
STXM: Scanning transmission X-ray microscopy
μXRF: Micro X-ray fluorescence microscopy
ICP-MS: Inductively coupled plasma-mass spectrometry
ROS: Reactive oxygen species
TEM: Transmission electron microscopy
MD: Molecular dynamics
DFT: Density-functional theory
PCM: Polarizable continuum model
IC50: Half maximal inhibitory concentration of a substance
sGO: Ultra-small graphene oxide
CBs: Nanocarbon blacks
EDS: Energy dispersive spectroscopy
DCFH-DA: Dichlorofluorescin diacetate
NAC: *N*-Acetylcysteine
7-AAD: 7-Amino-actinomycin
FBS: Fetal bovine serum
PBS: Phosphate buffered saline
SSRF: Shanghai Synchrotron Radiation Facility
