# Supplementary material for: Nanodiamonds act as Trojan horse for intracellular delivery of metal ions to trigger cytotoxicity
Source: Part Fibre Toxicol. 2015 Feb 5;12:2. doi: 10.1186/s12989-014-0075-z (PMC4374301; doi:10.1186/s12989-014-0075-z)
Supplement: Additional file 1: — Supplementary information. [file 12989_2014_75_MOESM1_ESM.doc]

Additional file 1

Supplementary information

**Methods**

***Theoretical calculations***

The most stable structures of ND particle at two possible carboxyl replaced site were obtained at the B3LYP/6-31+G(d,p) level, see Figure S1(a,b). The possible geometric structures of ND-metal ions (X=Cu^2+^, Ni^2+^, Cd^2+^ and Cr^3+^) are shown in Figure S1(c-h), which were obtained by using the B3LYP method combined with the 6-31+G(d,p) basis set for oxygen, carbon and hydrogen atoms and lanl2dz basis set for metal ions (Cu^2+^, Ni^2+^, Cd^2+^ and Cr^3+^). Table S1 shows their adsorption energies in water solution, which shows the most stable structure for Cu^2+^, Ni^2+^and Cr^3+^ on the ND particle is ND-X-1 (X=Cu^2+^, Ni^2+^and Cr^3+^), and the most stable structure for Cd^2+^ on the ND particle is ND-Cd^2+^-2.

The most stable structures of protonated ND particle (NDH) were obtained at the B3LYP/6-31+G(d,p) level, see Figure S8 (a,b). The possible stable structure of nanodiamond copper complex marked as NDH-Cu^2+^ is shown at the low pH in Figure S8 (c-h), which were obtained by using the B3LYP method combined with the 6-31+G(d,p) basis set for oxygen, carbon and hydrogen atoms and lanl2dz basis set for metal ions (Cu^2+^, Ni^2+^, Cd^2+^ and Cr^3+^). Table S2 shows their adsorption energies in water solution, which shows the most stable structure for Cu^2+^ on the NDH particle is NDH-Cu^2+^-1 (Figure S8c).

***Adsorption of Cu^2+^ on NDs***

Cu^2+^ solution (250 μg/mL) was mixed thoroughly with the aqueous ND solution (1 mg/mL). After incubation at 37 ºC for 1 min, 5 min, 15min, 30 min, 1 h, 2 h, 6 h and 12 h, mixtures were centrifuged at 12,000 rpm for 20 min, and the concentration of Cu^2+^ in the supernatant was determined by ICP-MS. The amounts of Cu^2+^ adsorbed on the NDs at certain time were obtained by subtraction. The variation of adsorption amounts with the elapsed times gave adsorption dynamics for the Cu^2+^ on NDs. By using the similar manner, the adsorption isotherm curves of Cu^2+^ on NDs (1 mg/mL and 50 μg/mL) at 37°C are obtained.

***Preparation and characterization of the ultra-small graphene oxide (sGO)***

Graphene oxide (GO) was prepared from purified natural graphite by using a modified Hummer’s method . To obtain the uniform size distributed ultra-small GO nanosheets (sGO), the as-prepared GO should be further oxidized. After oxidation, sGO was resuspended and dialyzed in millipore water for three days, and then sonicated for 2~3 h to obtain stable, well-dispersed sGO stock solutions.

A drop of medium solution (10 μL) was deposited onto a freshly-cleaved mica substrate for about 5 min. The substrate was then dried by a N_2_ stream. Atomic force microscopy (AFM, Nanoscope, Digital Co., USA) was used to observe the morphology of sGO. Results showed that the thickness of sGO nanosheets was at approximately 1.0 nm and their lateral dimensions ranged from 50 to 300 nm (see Figure 2a). UV-vis spectra showed that sGO dispersions displayed a maximum absorption at 218 nm, which is an identification measurement: maximum absorption peak at about 230 nm . FTIR spectrum of sGO showed the presence of O-H at 3463 cm^-1^, C=O at 1736 cm^-1^, C=C at 1636 cm^-1^, and C-O at 1100 cm^-1^. The details on chemical functionalization and characterization of the sGO will be described elsewhere.

***Nanocarbon blacks (CBs)***

Nanocarbon blacks (CBs) with mean aerodynamic diameter of 51 nm were obtained from Degussa (Shanghai, China). The total carbon content of CBs is greater than

98 wt%. TEM images showed that the size of the majority of CB clusters was about 90-250 nm (see Figure 2b). The details for characterization have been described in our previous work .

***Cytotoxicity assessment of Cu^2+^ and nanoparticle-Cu^2+^ mixture***

A L929 cell suspension (7×10^4^ cells/mL) was dispensed into 24-well plates and incubated overnight to allow for cell adherence. After washing twice with PBS, 50 μg/mL sGO, 50 μg/mL CBs, 25 μg/mL Cu^2+^, sGO-Cu^2+^ mixture, and CBs-Cu^2+^ mixture were added into the plate wells, respectively. Cells incubated with the complete culture medium were used as controls. Following 24 h incubation, cell aliquots were collected and immediately stained with trypan blue for 5 min. Cell proliferation was measured by counting cell numbers and cell viabilities were expressed as a percentage of Number_test_/Number_control_.

***Molecular dynamics computational methods***

The initial simulation box size is Lx = 6.000 nm, Ly = 6.000 nm and Lz = 6.000 nm. Periodic boundary conditions are applied in all directions. During the simulations, we fix the ND or NDH particle, which contains 126 carbon atoms and 8 oxygen atoms, 82 (ND) or 86 (NDH) hydrogen atoms, respectively, and the geometry structures come from the density-functional calculations (at the B3LYP/6-31G(d) level), as implemented in the Gaussian 09 program. There are 8 ND or NDH particles, 160 Cu^2+^, 320 Cl^-^ ions (another 32 Na^+^ ions are added to retain the system charge neutral in ND particle system) together with 6214 water molecules in this simulation system and all molecules and ions are free to move in this simulation. A constant-temperature (300 K) and constant-volume molecular dynamics simulation is performed using a time step of 1.0 fs with Gromacs 4.5.4. All of the atoms and ions parameters are taken from the OPLSAA force field and the SPC/E water model is used. The simulation time is 4 ns for ND or NDH with Cu^2+^ solutions. The selection of a vapor-liquid coexistence system is used to maintain the ambient condition , and a Berendsen thermostat with a time constant of 1.0 ps for coupling is used to maintain the temperature. The particle-mesh Ewald method with a real-space cut-off of 10 Å is used to model long-range electrostatic interactions, and the same 10 Å cut-off is applied to the van der Waals interactions.

**References**

1. Hummers WS, Offeman RE: **Preparation of Graphitic Oxide.** *J Am Chem Soc* 1958, **80:**1339-1339.

2. Shan CS, Yang HF, Han DX, Zhang QX, Ivaska A, Niu L: **Water-Soluble Graphene Covalently Functionalized by Biocompatible Poly-L-lysine.** *Langmuir* 2009, **25:**12030-12033.

3. Luo DC, Zhang GX, Liu JF, Sun XM: **Evaluation Criteria for Reduced Graphene Oxide.** *J Phys Chem C* 2011, **115:**11327-11335.

4. Kong HT, Zhang Y, Li YJ, Cui ZF, Xia K, Sun YH, Zhao QF, Zhu Y: **Size-Dependent Cytotoxicity of Nanocarbon Blacks.** *Int J Mol Sci* 2013, **14:**22529-22543.

5. Hess B, Kutzner C, van der Spoel D, Lindahl E: **GROMACS 4: Algorithms for highly efficient, load-balanced, and scalable molecular simulation.** *J Chem Theory Comput* 2008, **4:**435-447.

6. Berendsen HJC, Grigera JR, Straatsma TP: **The Missing Term in Effective Pair Potentials.** *J Phys Chem* 1987, **91:**6269-6271.

7. Koishi T, Yoo S, Yasuoka K, Zeng XC, Narumi T, Susukita R, Kawai A, Furusawa H, Suenaga A, Okimoto N, et al: **Nanoscale hydrophobic interaction and nanobubble nucleation.** *Phys Rev Lett* 2004, **93**.

8. Darden T, York D, Pedersen L: **Particle Mesh Ewald - an N.Log(N) Method for Ewald Sums in Large Systems.** *J Chem Phys* 1993, **98:**10089-10092.

**Supplementary movies, figures and tables**

**Movies of Cu^2+^ adsorption on the ND (ND-Cu^2+^) and NDH (NDH-Cu^2+^) particles**


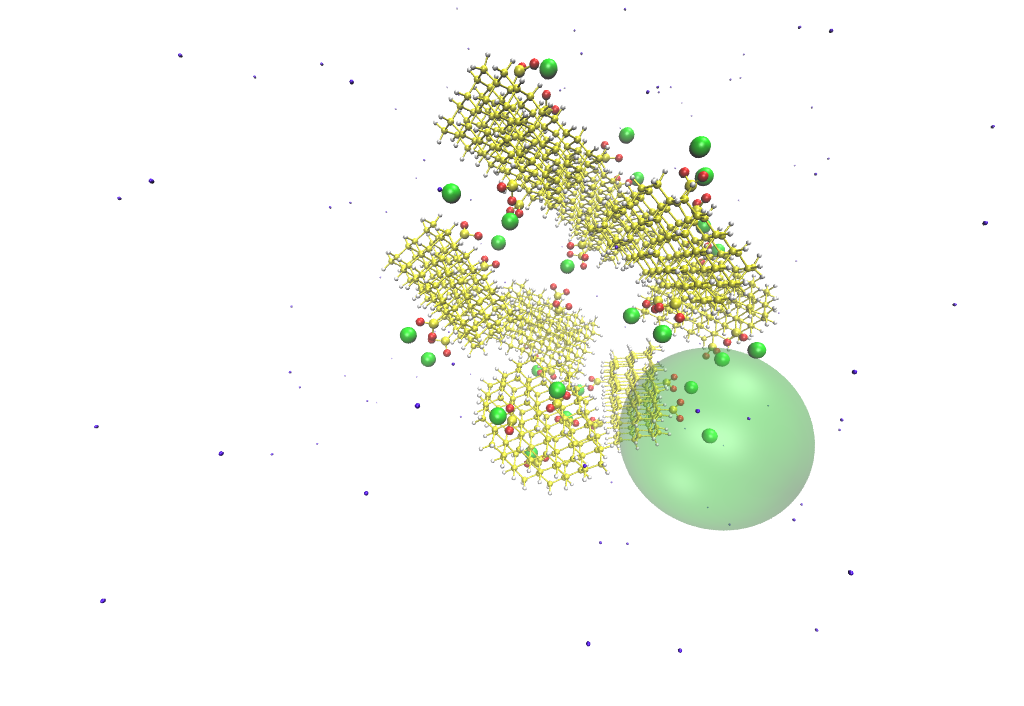

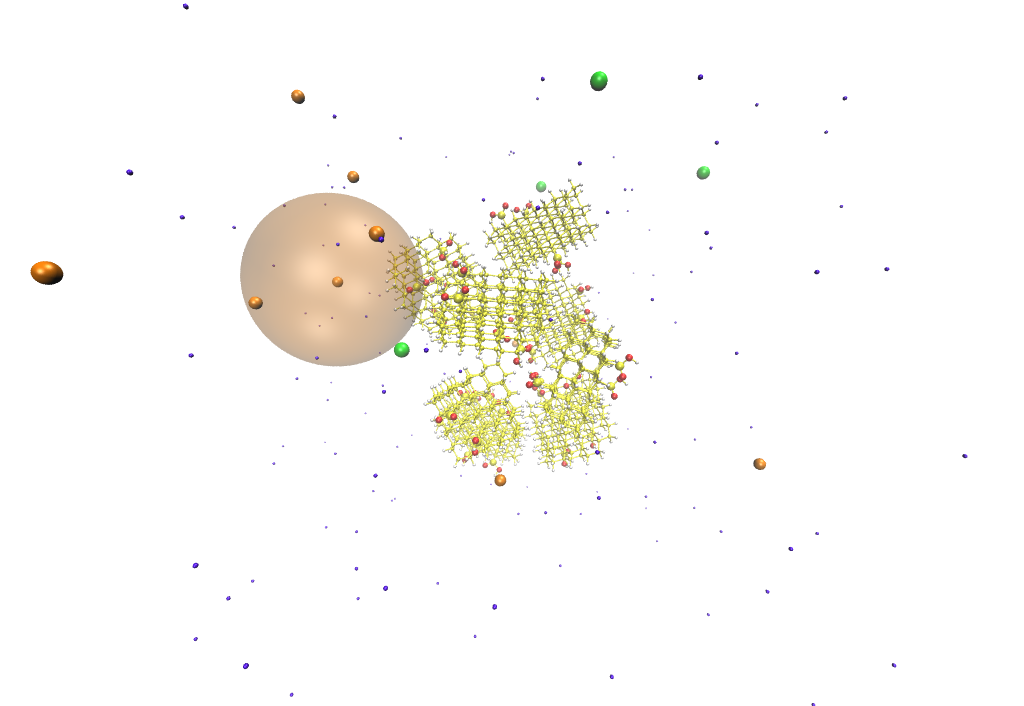


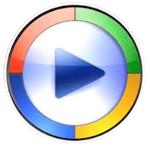

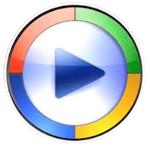


**ND-Cu^2+^**

**NDH-Cu^2+^**

ND particle are shown with carbon in yellow, hydrogen in white and oxygen in red. Copper ions are shown in blue (out the ND or NDH adsorption distance), green (in the ND or NDH adsorption distance) and orange (from the NDH adsorption to freedom distribution in water), respectively. A green ball with “green balloon (the adsorption distance of copper ions on the ND)” in **ND-Cu^2+^** showed the adsorption process of copper ion from freedom distribution in water to the ND adsorption. An orange ball with “orange balloon (the adsorption distance of copper ions on the NDH)” in **NDH-Cu^2+^** showed the leaving process of copper ion from the NDH adsorption distance to freedom distribution in water.


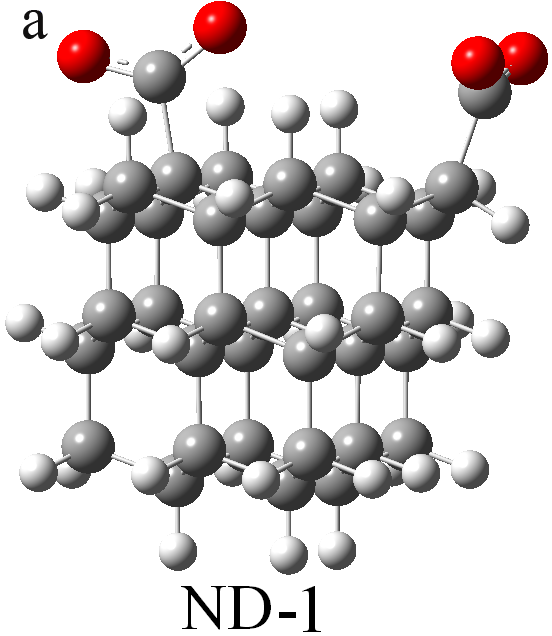

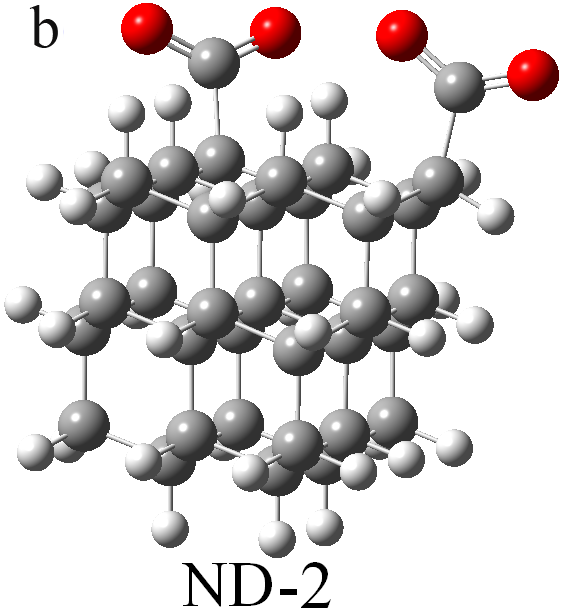

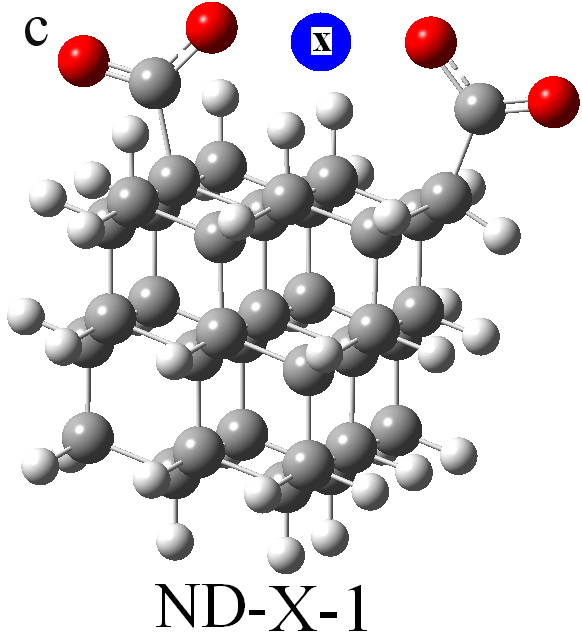

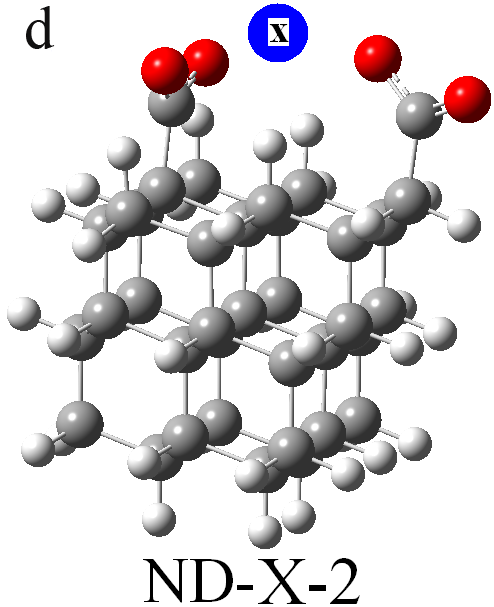


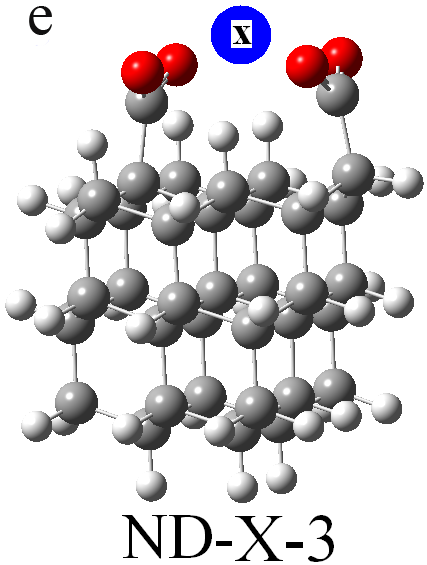

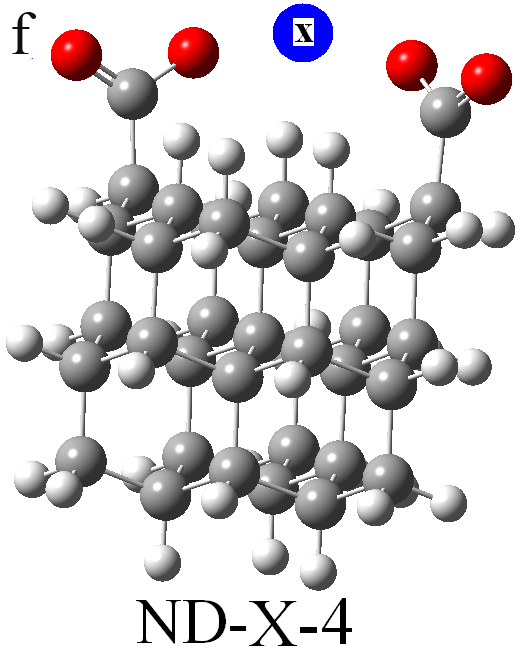

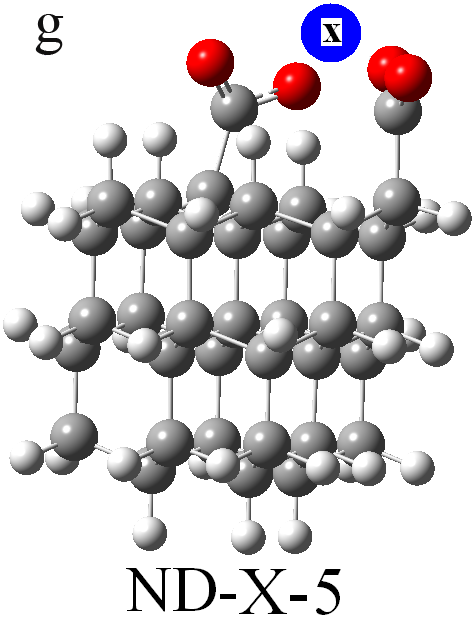

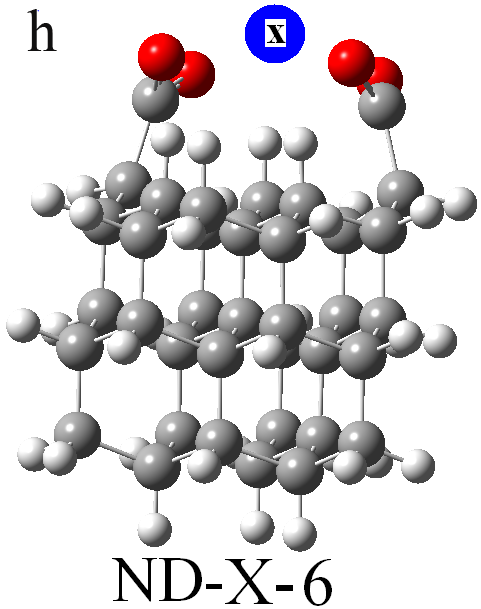


**Figure S1. ND particle and ND-ion complex structures. a,b,** The most stable structures of ND particle at two possible carboxyl replaced site. **c-h,** The most stable structures of ND-ion complexes (X=Cu^2+^, Ni^2+^, Cd^2+^ and Cr^3+^).

**a**

**b**

**
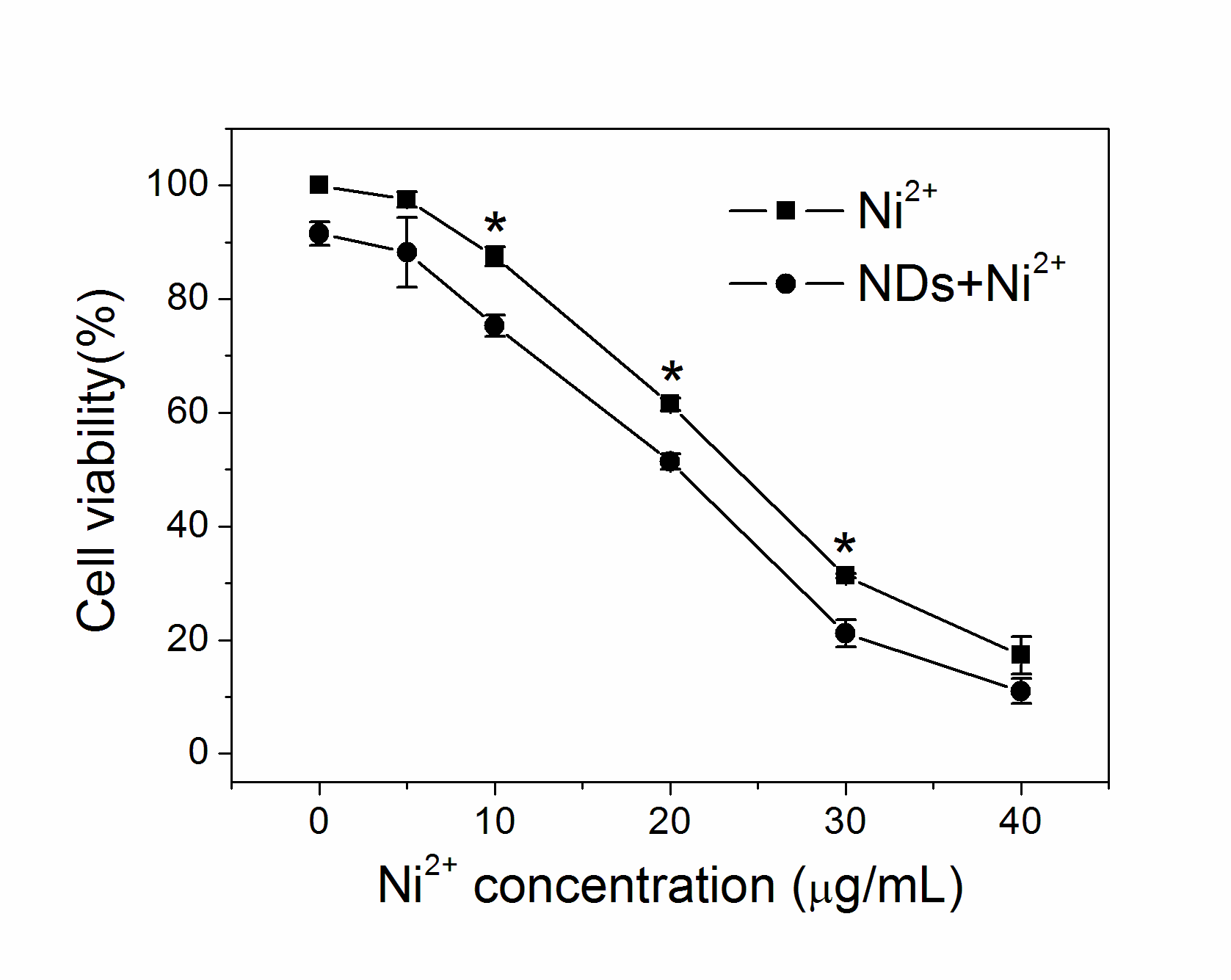
**


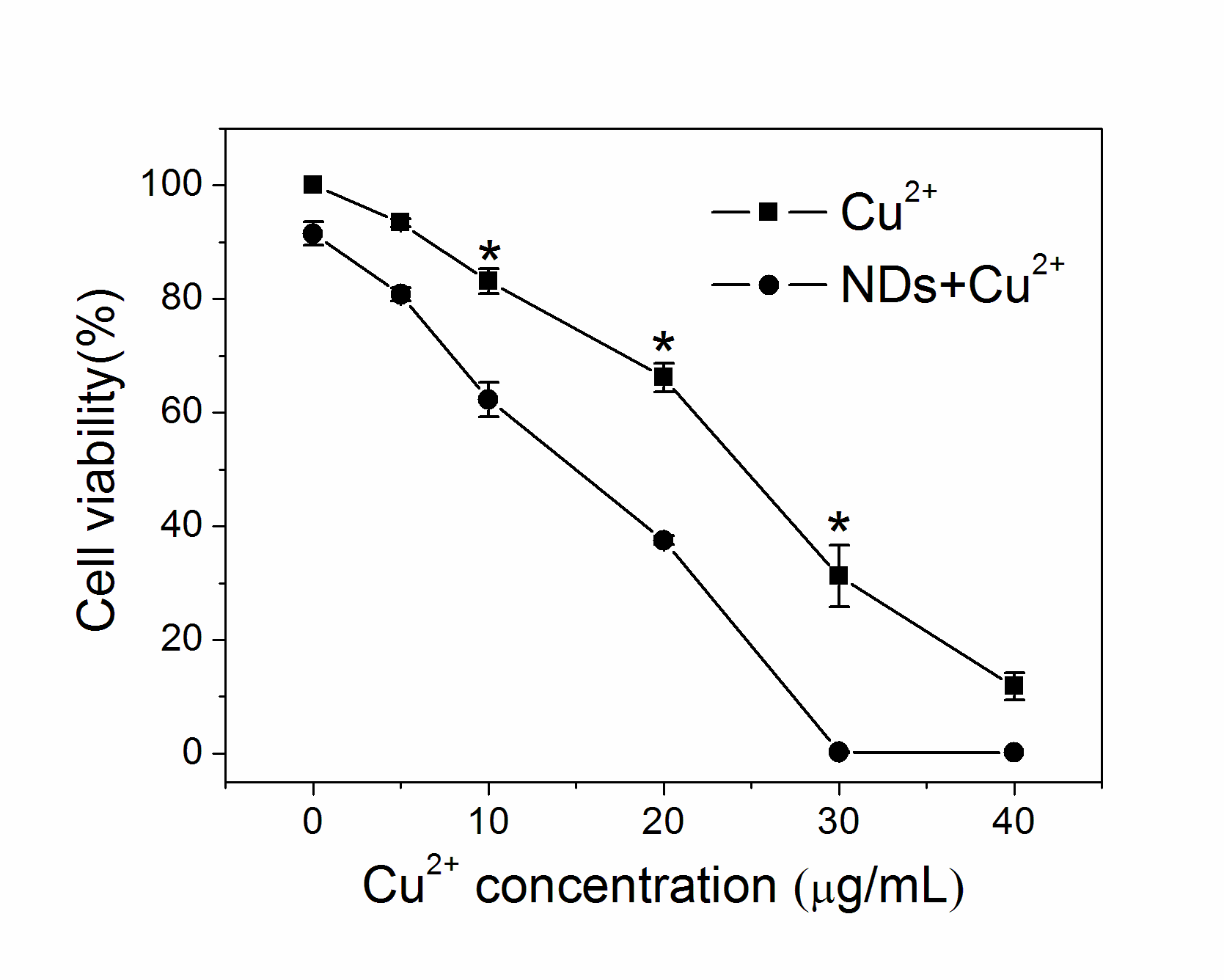


**c**

**d**


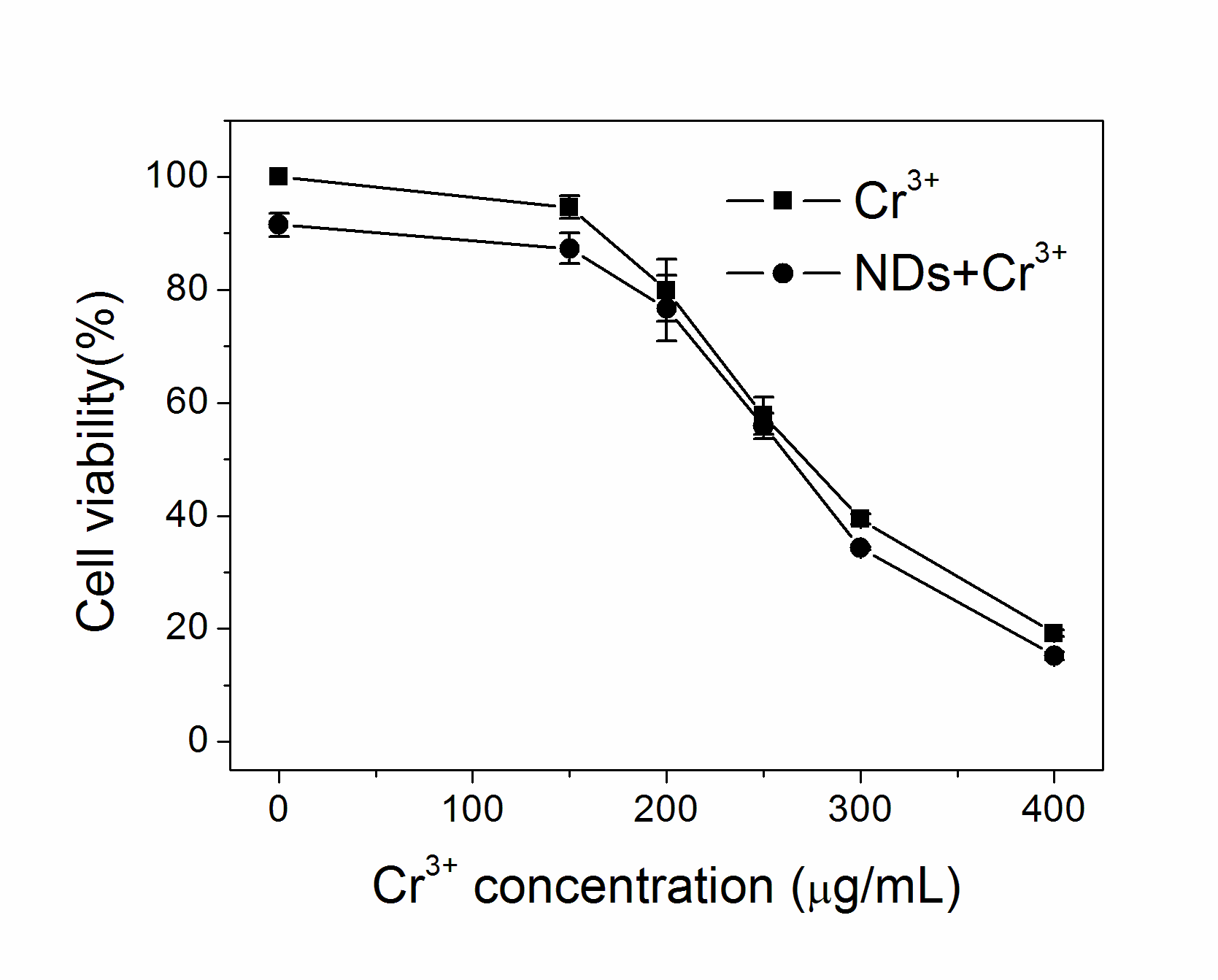

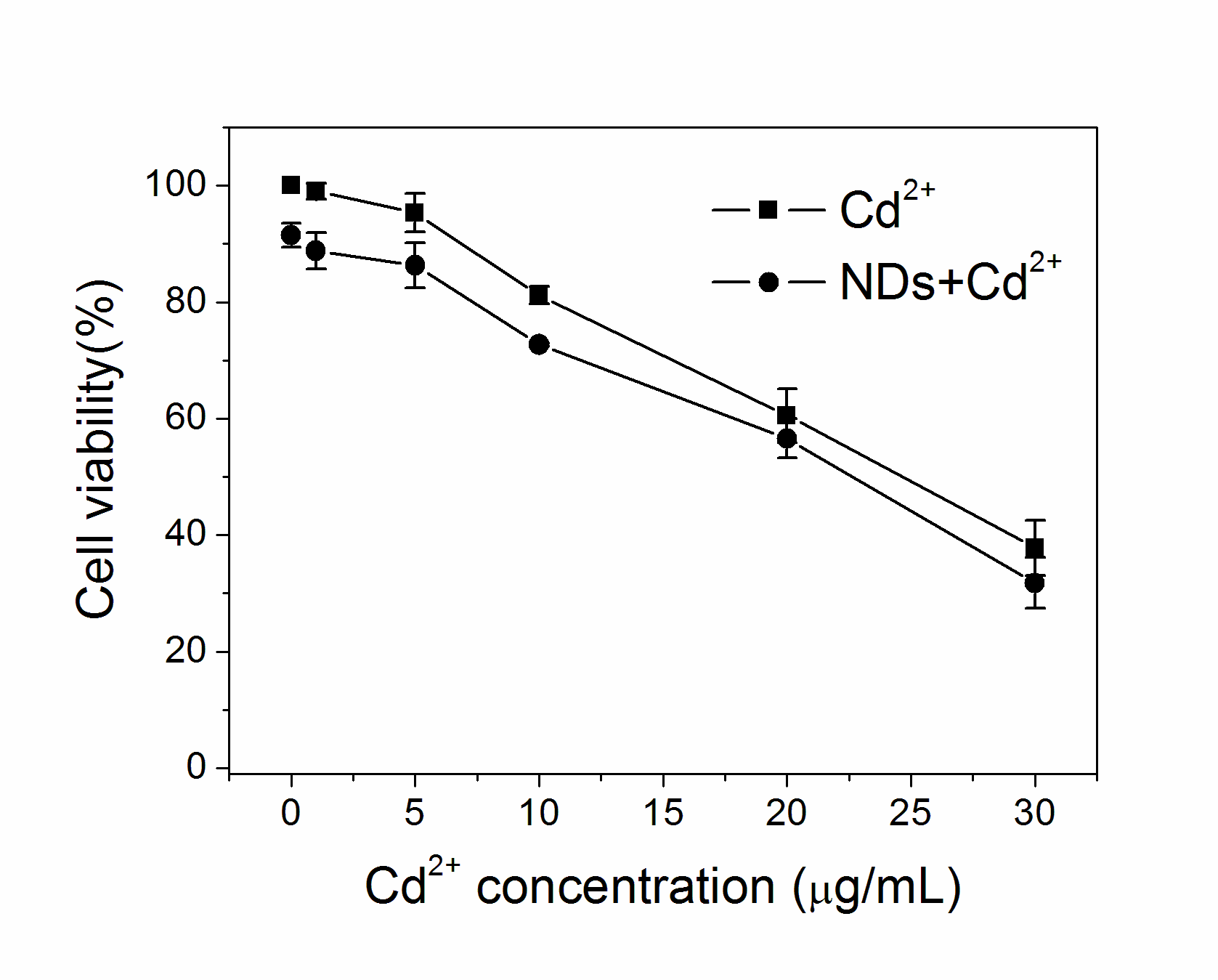


**Figure S2. The differential viability of L929 in individual metal ion at various concentration with or without 50 μg/mL NDs after treatment for 24 h (*N*=3; error bars are SD)**. **a,** Cu^2+^ at various concentration ranging from 0 to 40 μg/mL, **b,** Ni^2+^ at various concentration ranging from 0 to 40 μg/mL, **c,** Cd^2+^ at various concentration ranging from 0 to 30 μg/mL, **d,** Cr^3+^ at various concentration ranging from 0 to 400 μg/mL (*p＜0.05, *t*-tests for comparison).

**a**

**b**


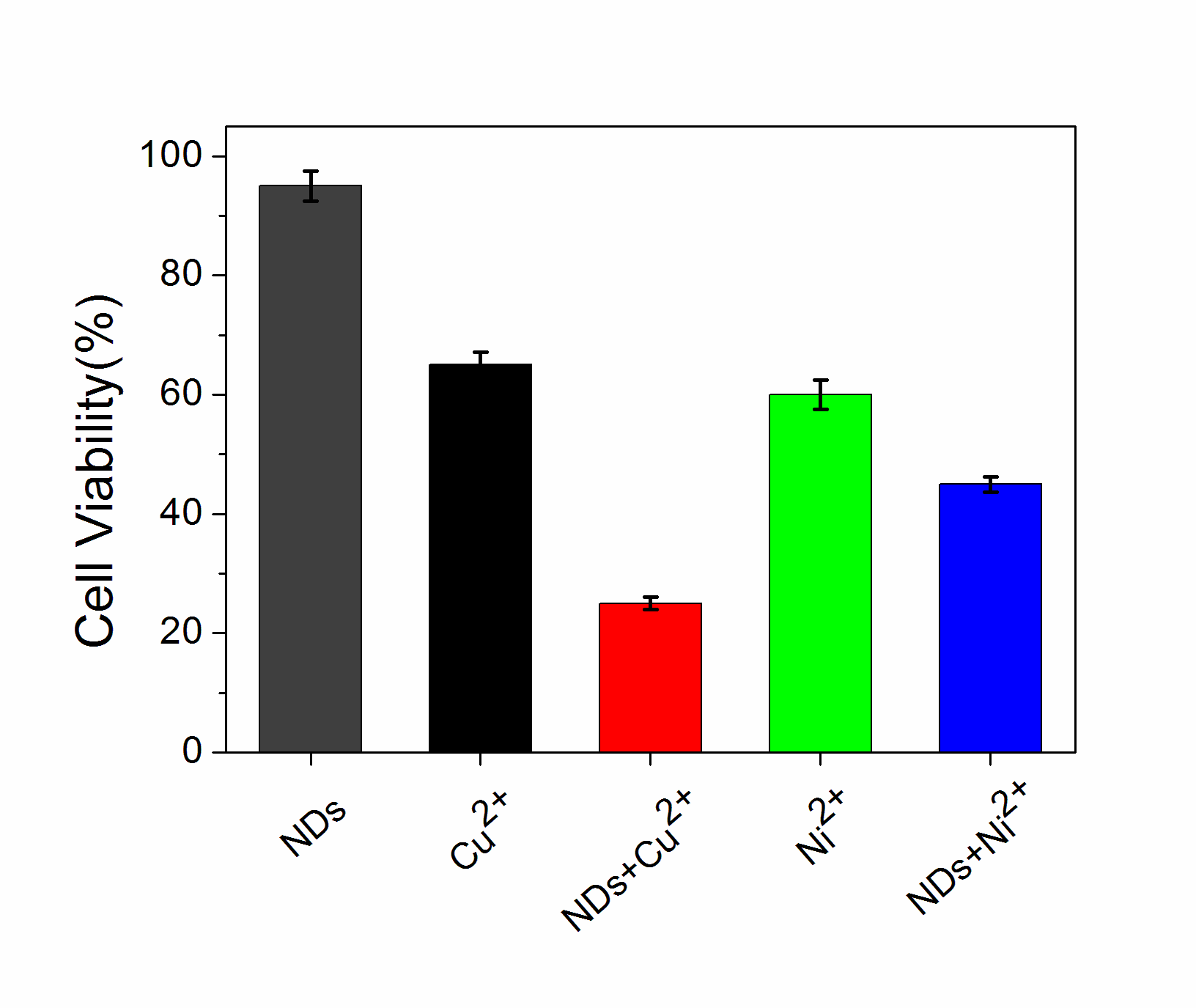

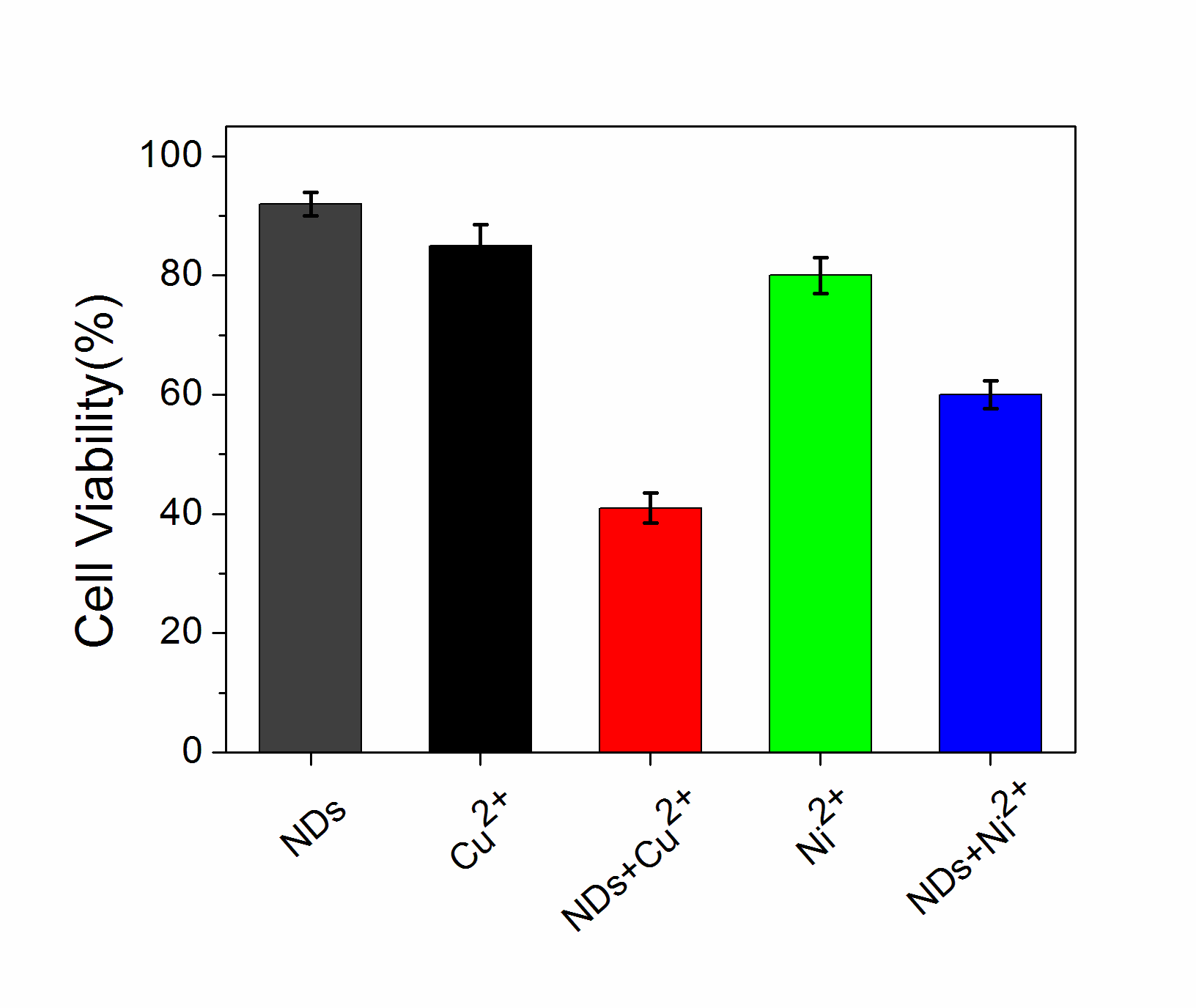


**Figure S3. The differential cell viability in metal ions with or without 50 μg/mL NDs after treatment for 24 h (*N*=3; error bars are SD)**. **a,** BEAS-2B cells, **b,** HaCaT cells. The concentration of metal ions is 25 μg/mL.


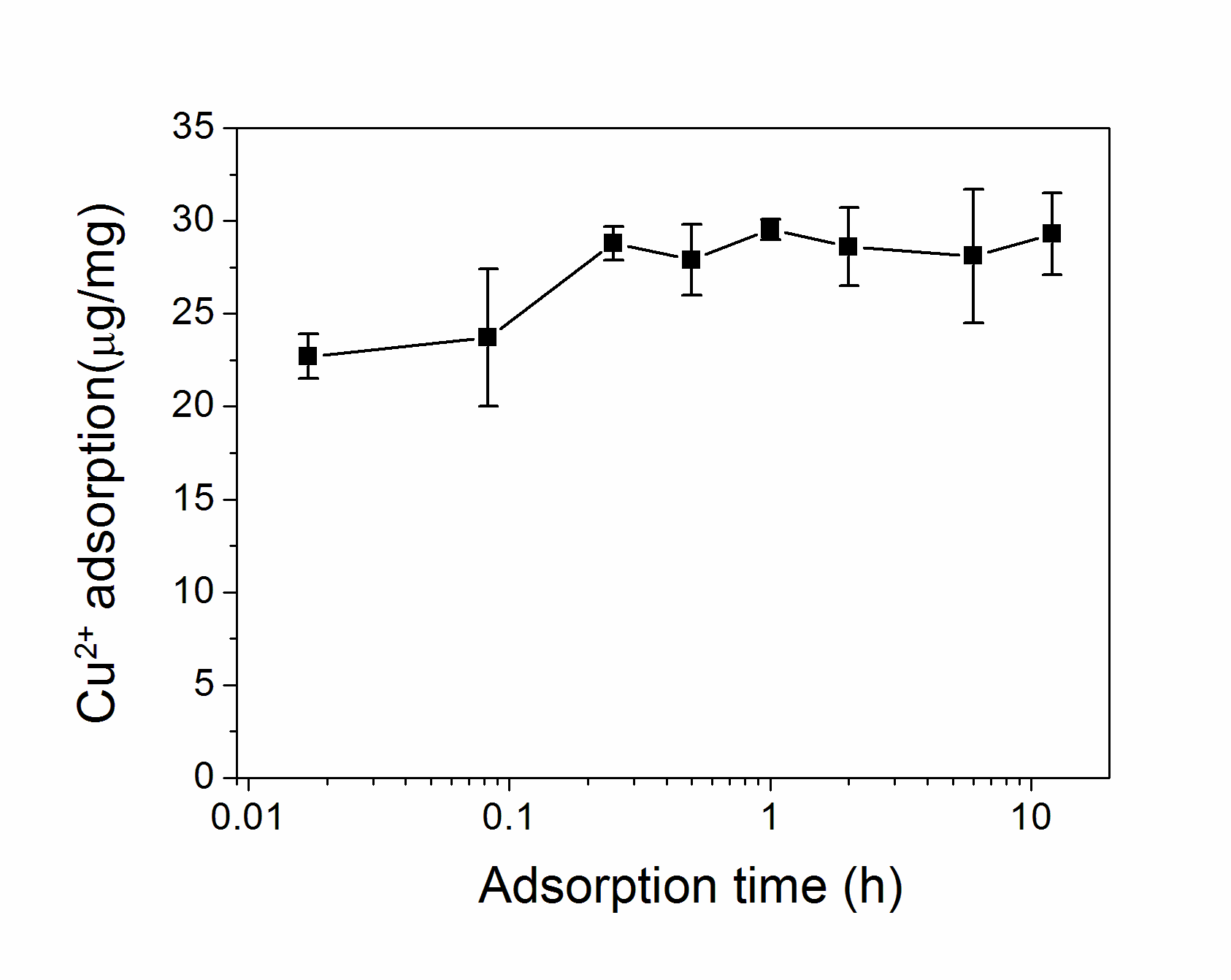


**a**


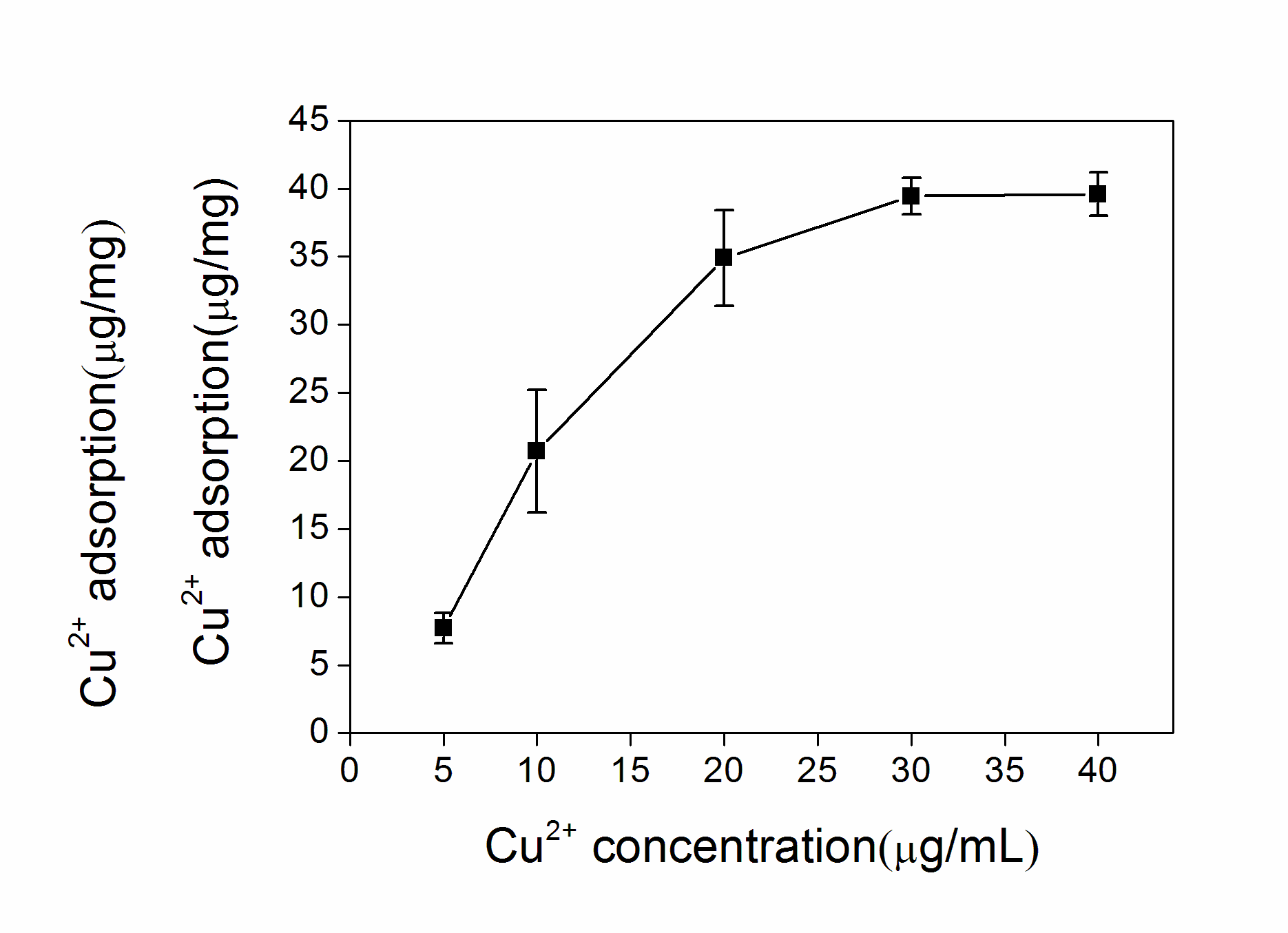


**b**

**c**

**
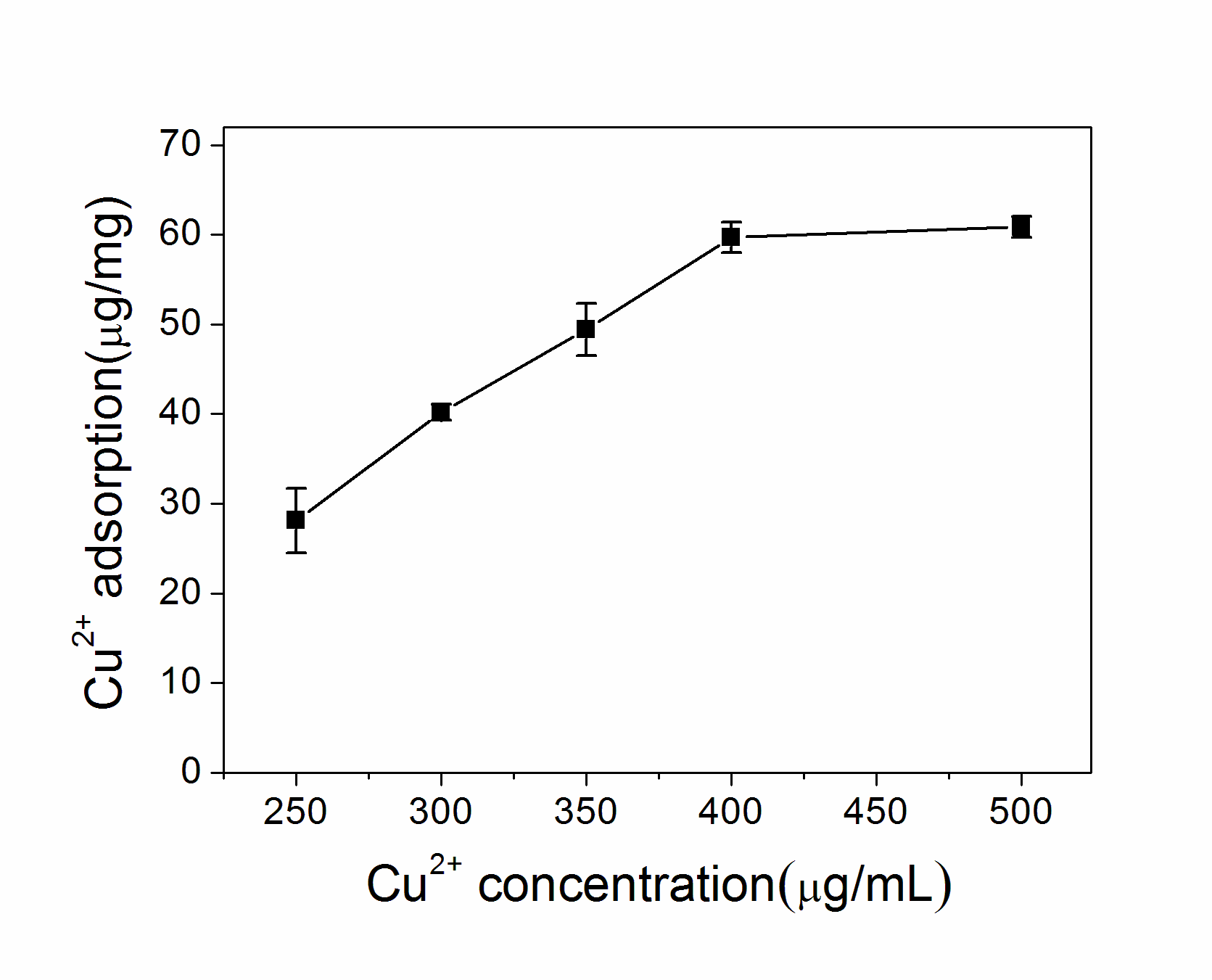
**

**Figure S4. Adsorption of Cu^2+^ on NDs**. **a,** The adsorption kinetics curves of Cu^2+^ on NDs. The concentration of NDs is 1 mg/mL. **b, c**, The adsorption isotherm curves of Cu^2+^ on NDs. The concentration of NDs is 1mg/mL (**b**) and 50 μg/mL (**c**).


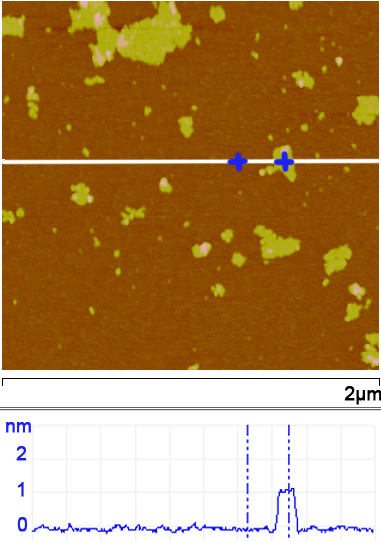


**a**

**b**

**c**

**
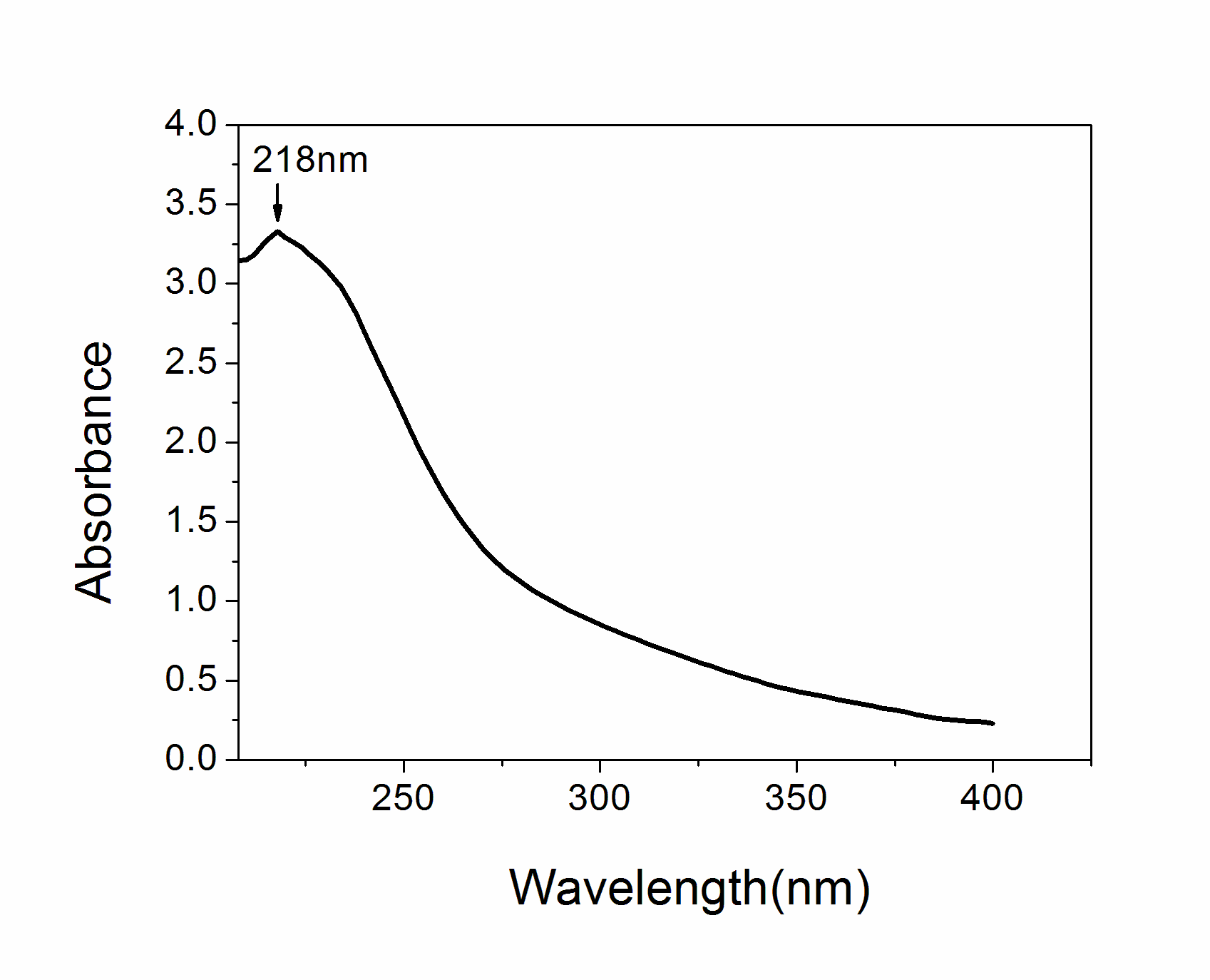

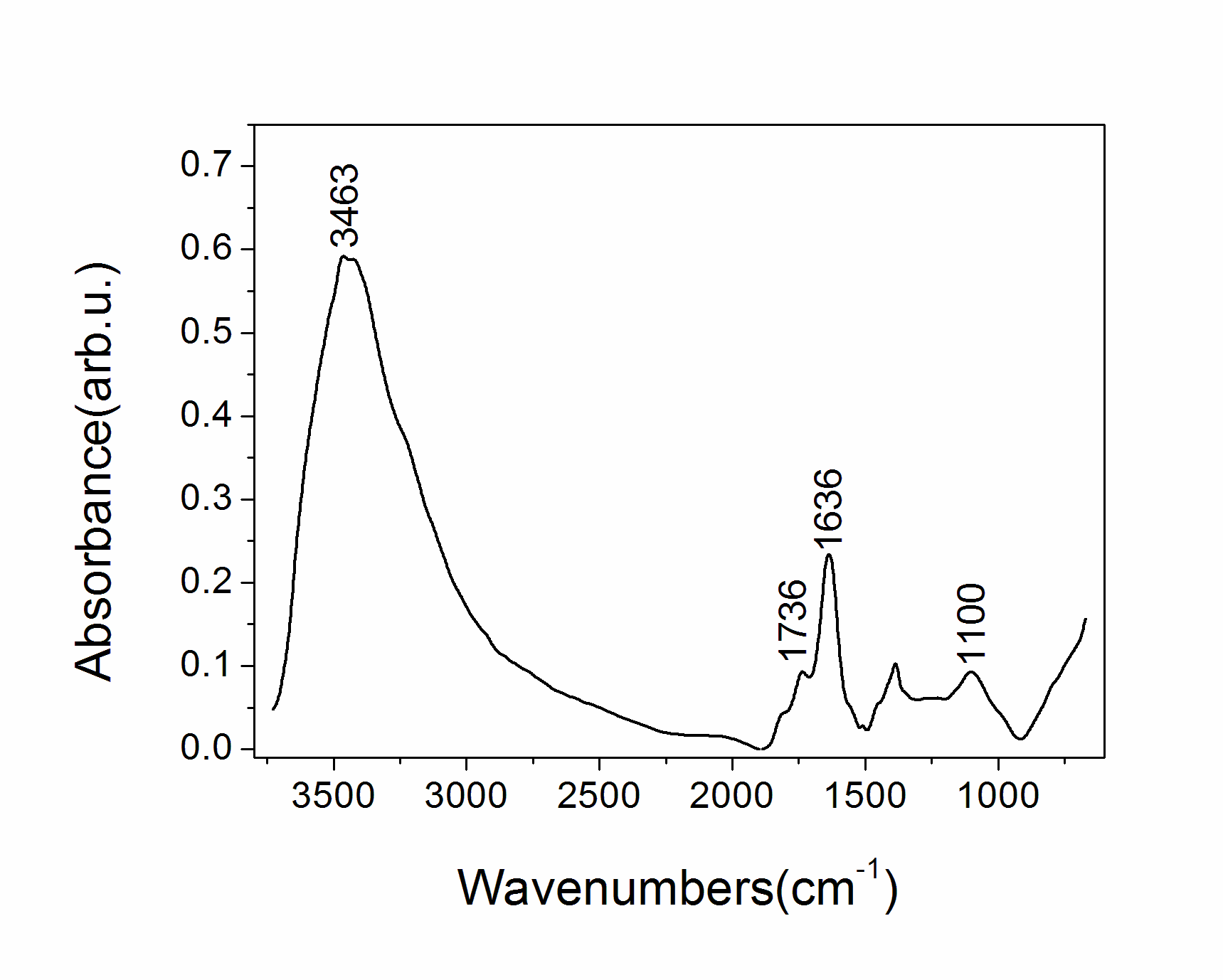
**

**Figure S5. Characterization of sGO.** **a,** AFM images of sGO. **b,** UV-vis spectra of sGO. **c,** FTIR spectra of sGO.


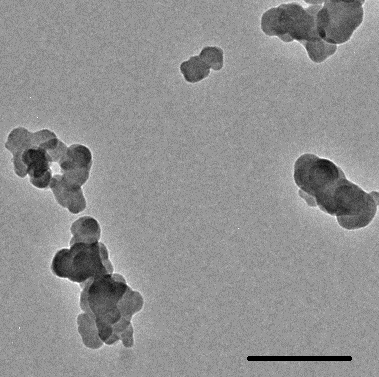


**200nm**

**Figure S6. TEM image of CBs.**


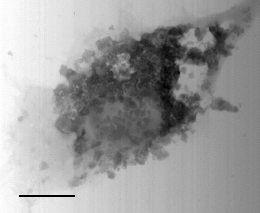


**934 eV NDs**


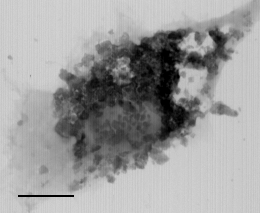


**936.6eV NDs**

**c**

**d**


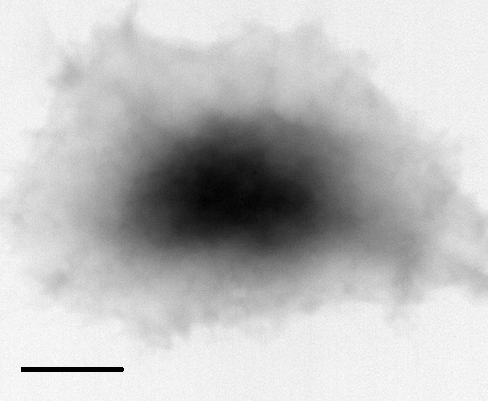


**934 eV** **Cu^2+^**


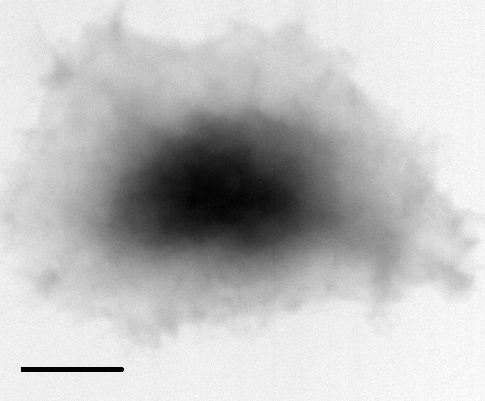


**936.6eV Cu^2+^**

**e**

**f**


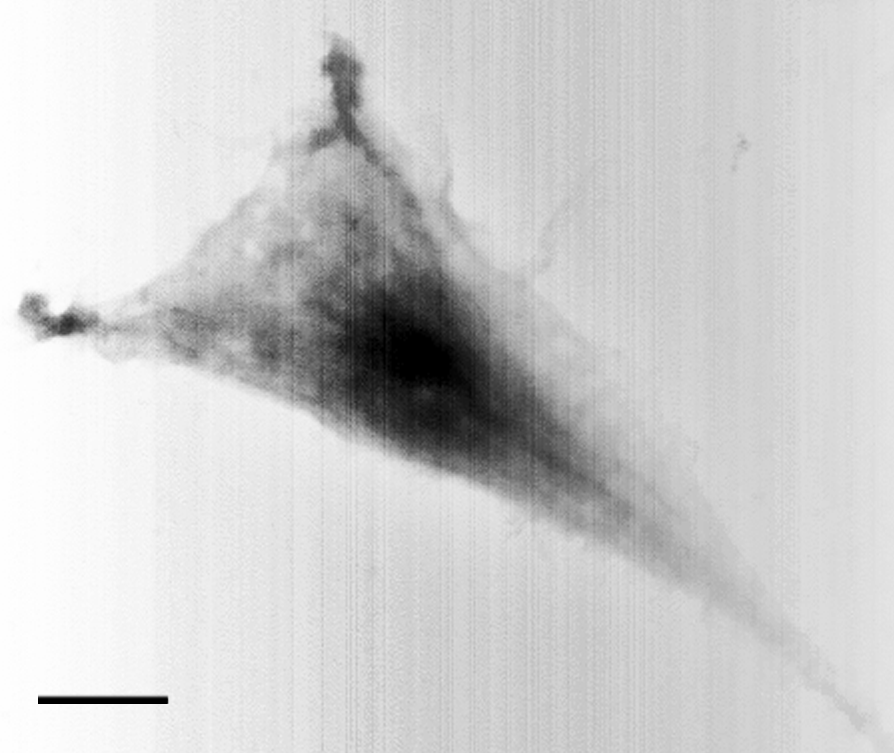


**934 eV control**


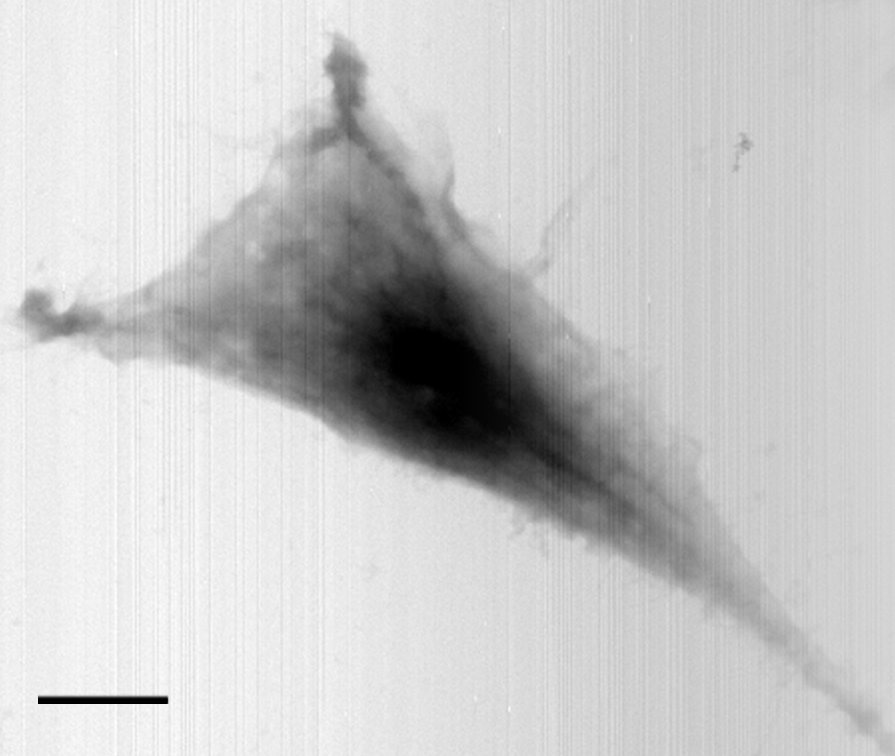


**936.6eV control**

**a**

**b**

**5μm**


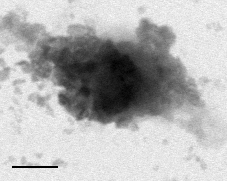


**934 eV ND-Cu^2+^**

**g**


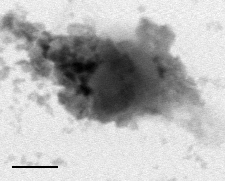


**936.6eV ND-Cu^2+^**

**h**

**Figure S7.** **Absorption-contrast images of a typical L929 cell at E1 = 936.6 eV and E2 = 934 eV.** **a, b,** control, **c-h**, cells after incubation with NDs (**c, d**), Cu^2+^(**e, f)**, and ND-Cu^2+^ mixture (**g, h**). The step is 100 nm for **(a, b, g, h**), 150 nm for (**c, d**), and 50 nm for (**e, f**).


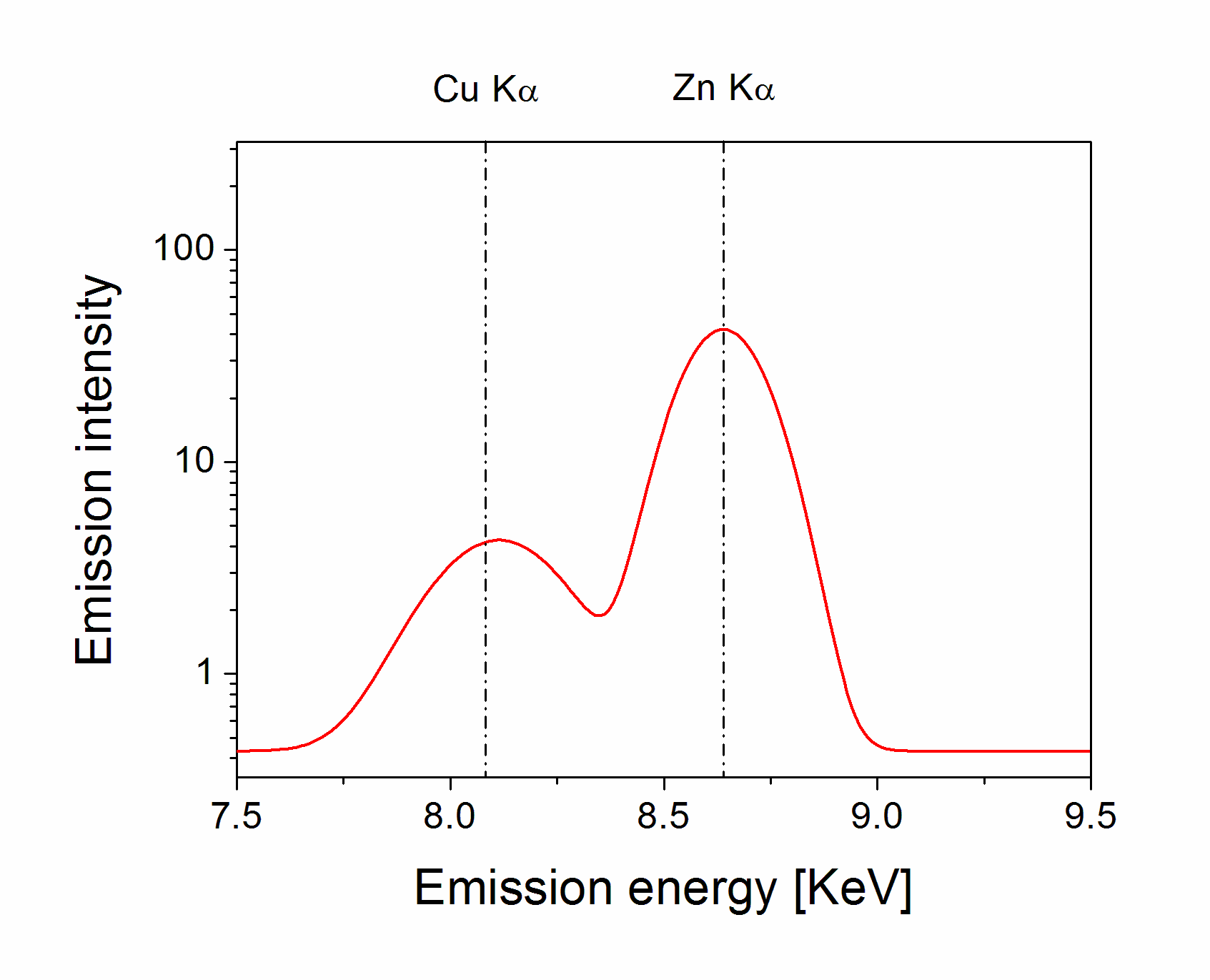


**a**


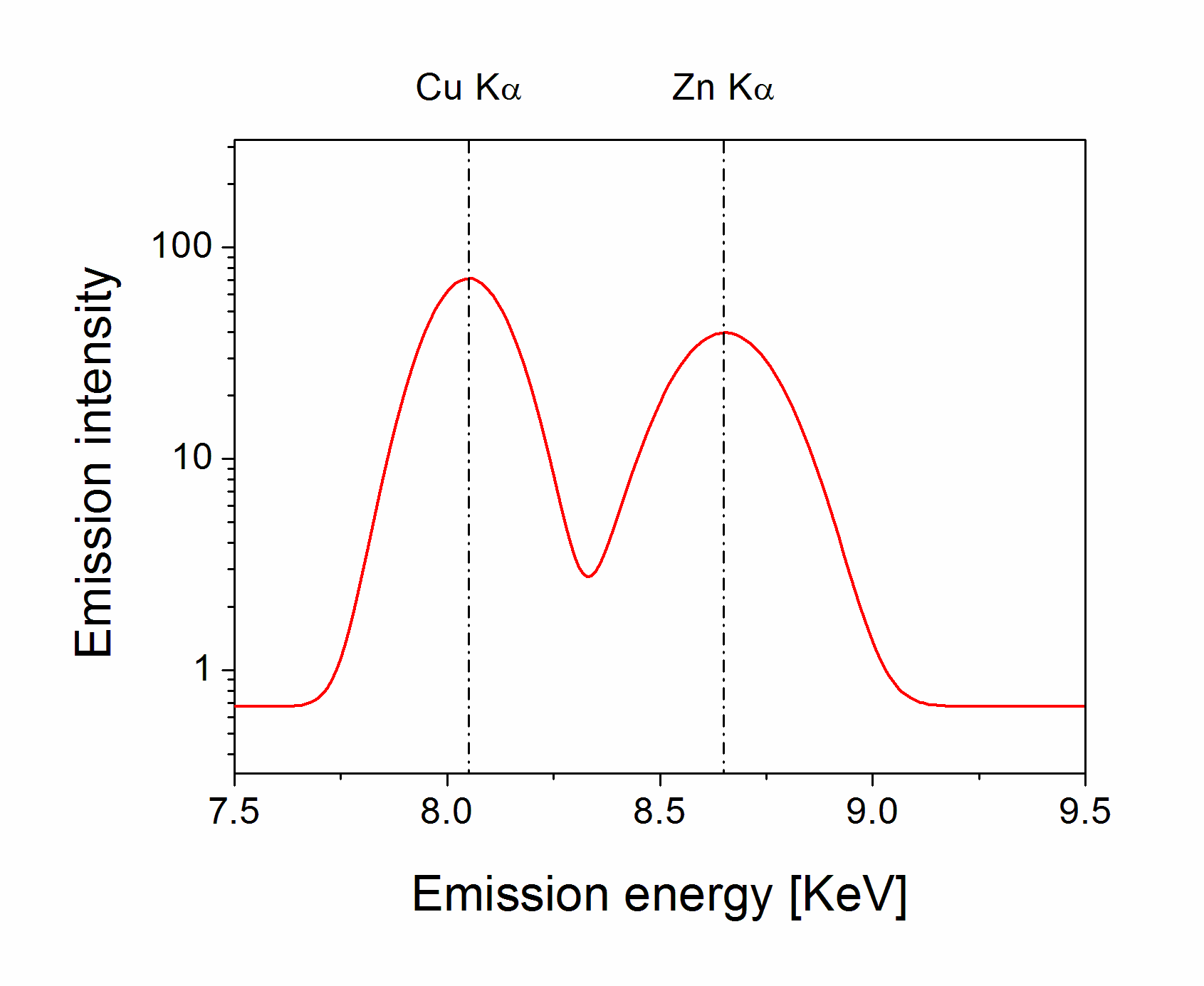


**b**


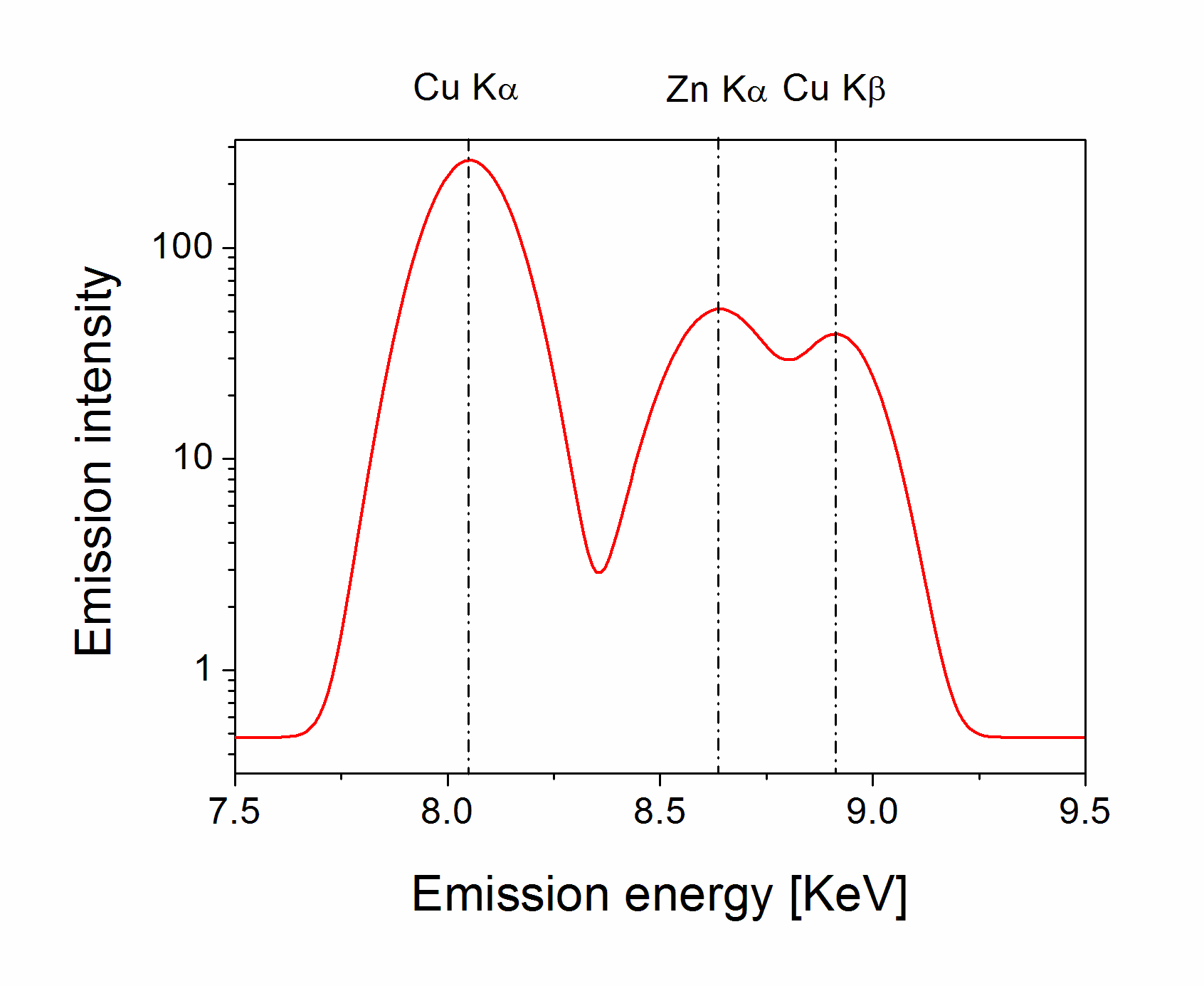


**c**

**Figure S8. X-ray emission spectra for copper and zinc averaged over the entire area of the L929 cells (normalized to beam intensity).** **a-c**, cells after incubation with basal medium or NDs (**a**), Cu^2+^ (**b**), and ND-Cu^2+^ mixture (**c**) for 24 h.


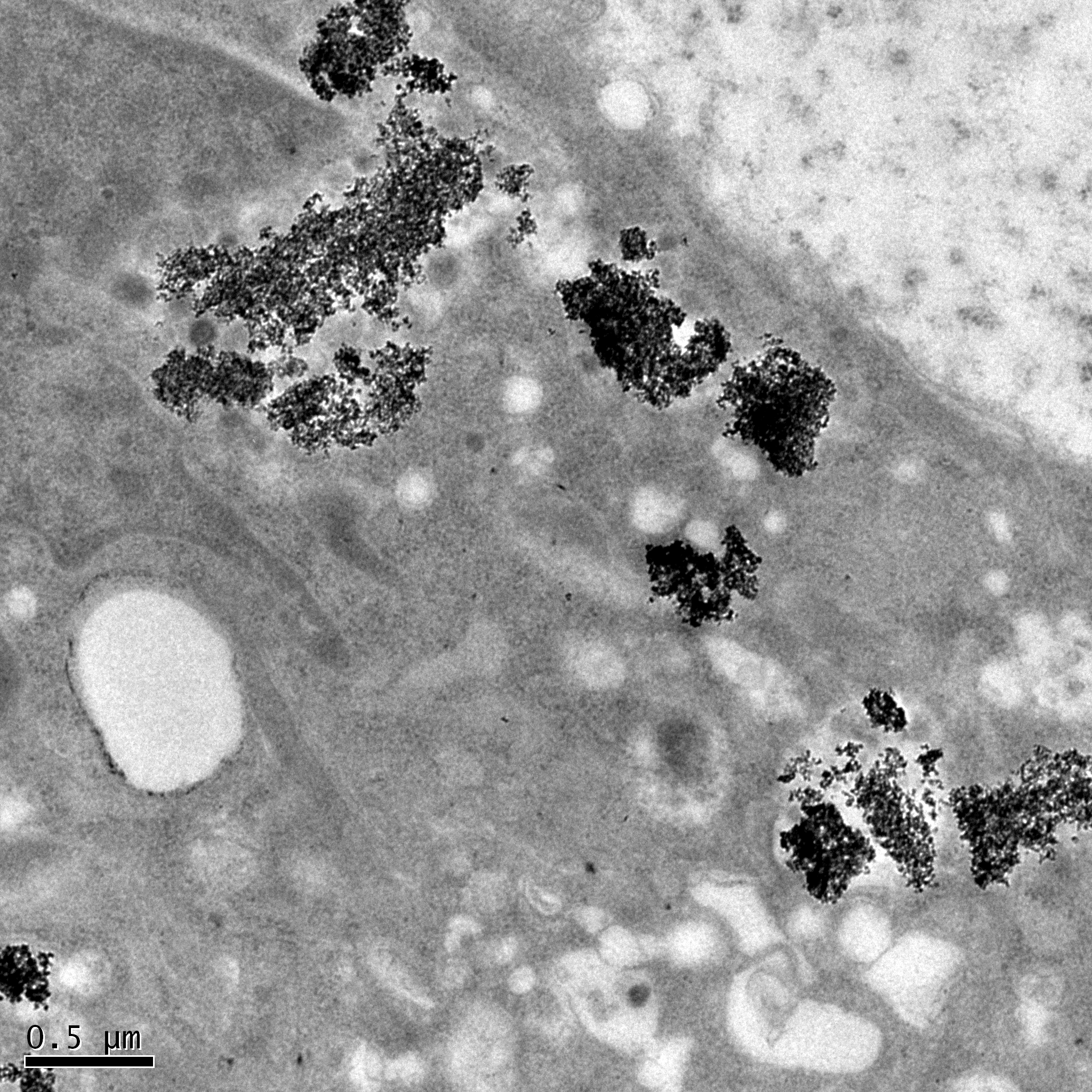

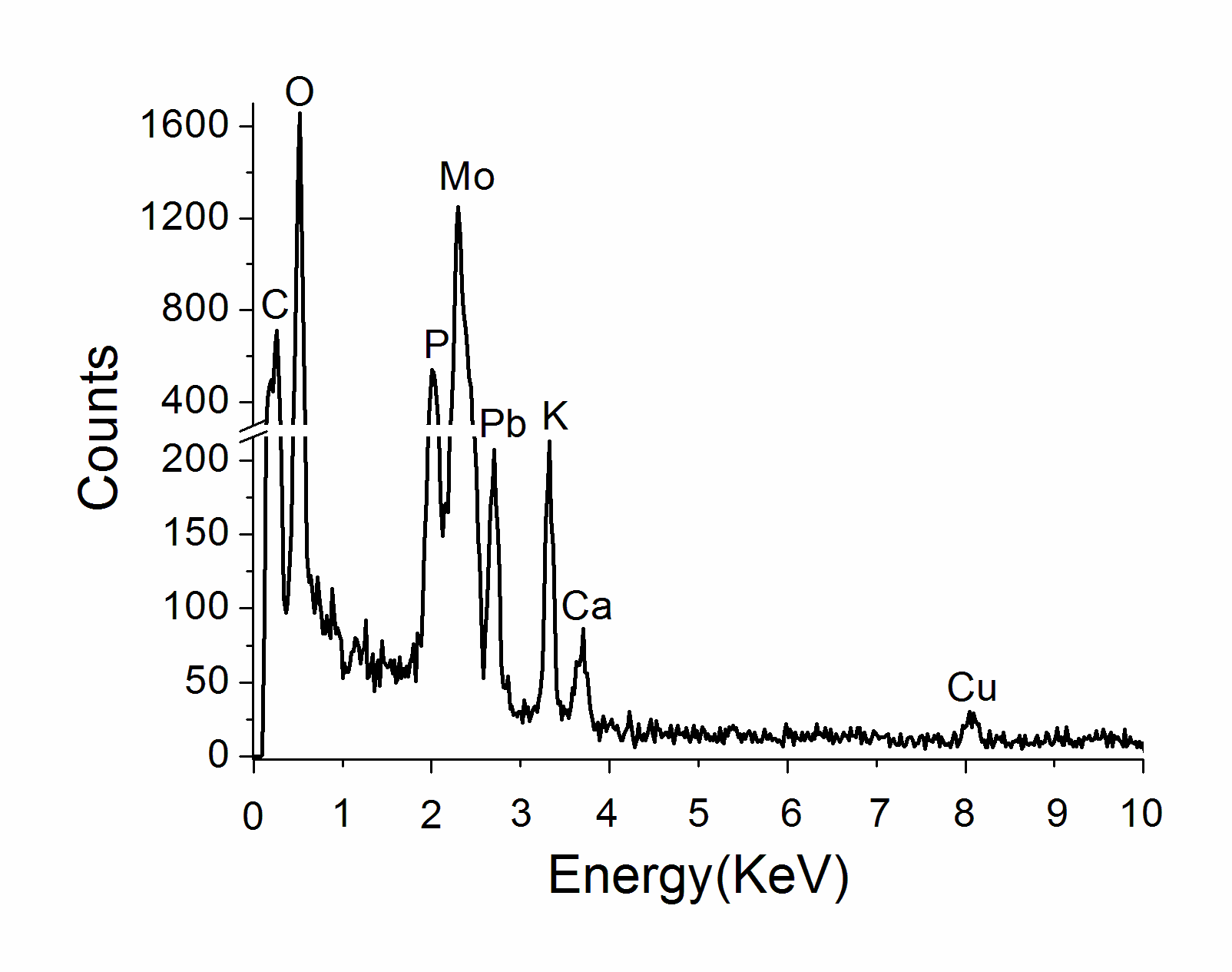


**a**

**b**

**Figure S9. TEM image of cells after incubation with ND-Cu^2+^ mixture (scale bar: 0.5**μ**m) (a) and energy dispersive spectroscopy (EDS) microanalysis of intracellular ND aggregates, showing the presence of Cu (b).** Sample was placed on a molybdenum TEM grid for examination.


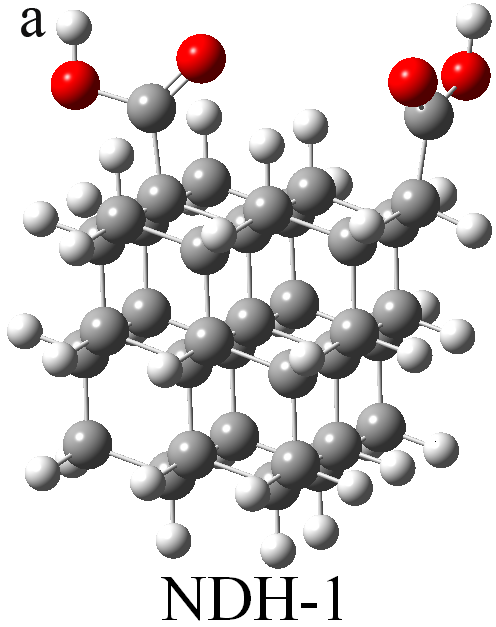

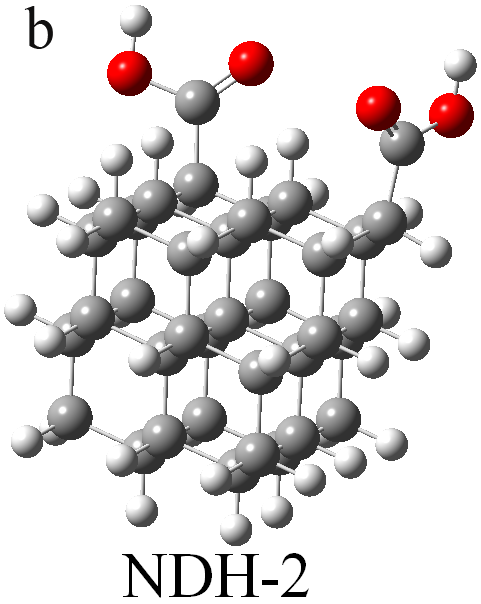

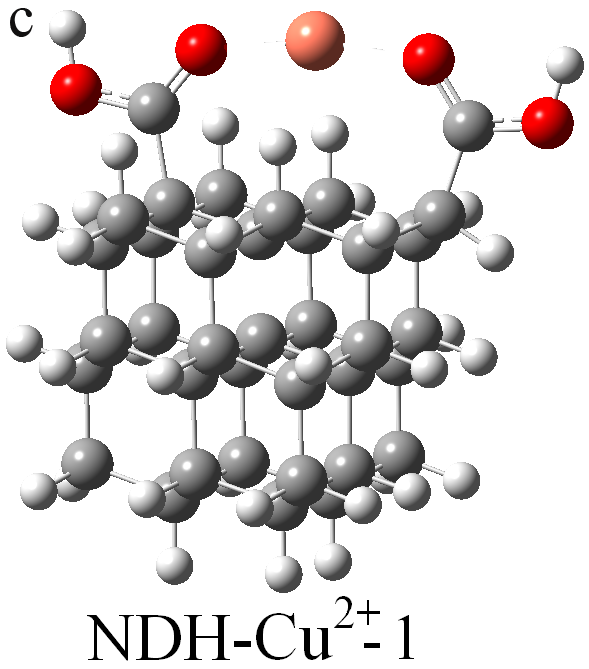

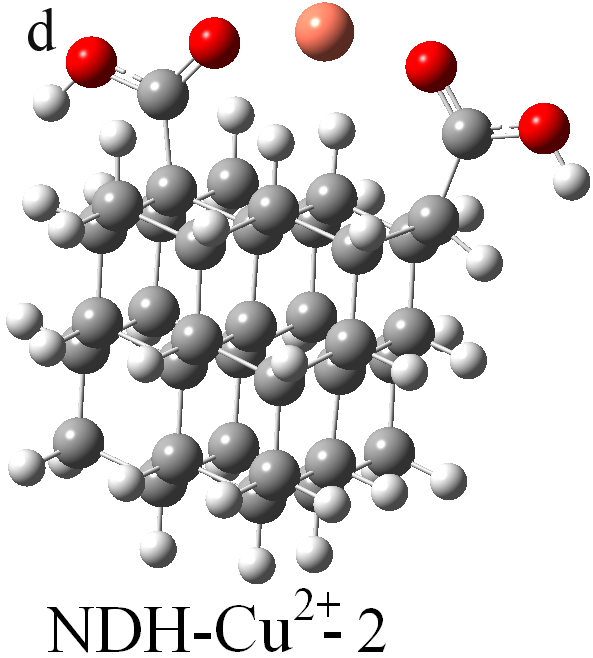


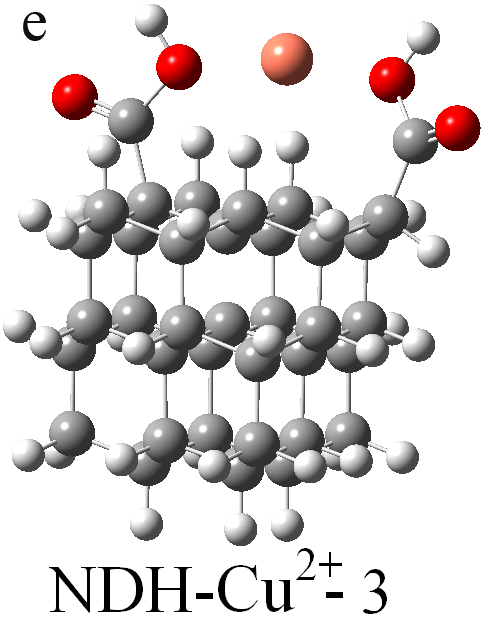

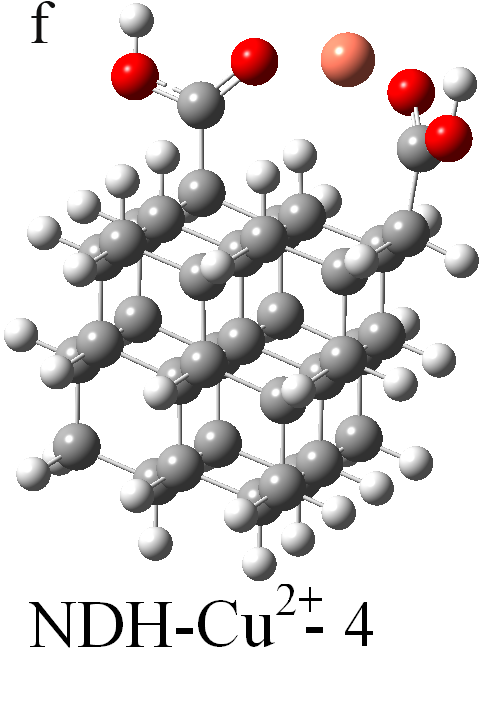

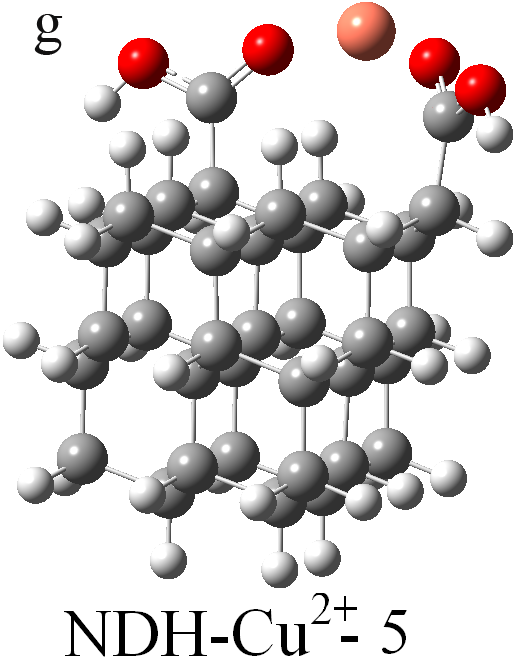

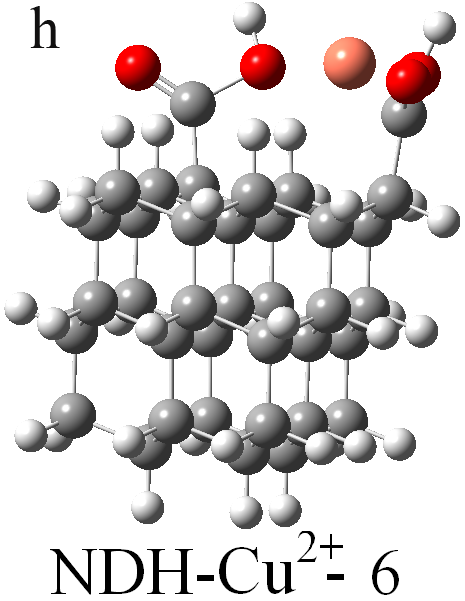


**Figure S10. Protonated ND particle (NDH) and NDH-Cu^2+^ complex structures. a, b,** The most stable structures of NDH at two possible carboxyl replaced site. **c-h,** The possible stable structures of the NDH-Cu^2+^ complexes.

**Table S1.** **The adsorption energies of the possible geometric structures of ND-ion complexes (X=Cu^2+^, Ni^2+^, Cd^2+^ and Cr^3+^) in water solution.**

| ND-ion complexes | ΔE_i_ | ND-ion complexes | ΔE_i_ |
| --- | --- | --- | --- |
| ND-1+X  (X=Cu^2+^, Ni^2+^, Cd^2+^, Cr^3+^) | 0 | ND-2+X  (X=Cu^2+^, Ni^2+^, Cd^2+^, Cr^3+^) | 0 |
| ND+Cu^2+^-1 | -7.38 | ND+Cu^2+^-4 | -6.76 |
| ND+Cu^2+^-2 | -6.39 | ND+Cu^2+^-5 | / |
| ND+Cu^2+^-3 | -6.10 | ND+Cu^2+^-6 | -5.80 |
| ND+Ni^2+^-1 | -2.93 | ND+Ni^2+^-4 | -2.49 |
| ND+Ni^2+^-2 | -2.50 | ND+Ni^2+^-5 | -2.18 |
| ND+Ni^2+^-3 | / | ND+Ni^2+^-6 | / |
| ND+Cd^2+^-1 | -0.31 | ND+Cd^2+^-4 | -0.64 |
| ND+Cd^2+^-2 | -0.71 | ND+Cd^2+^-5 | -0.34 |
| ND+Cd^2+^-3 | -0.08 | ND+Cd^2+^-6 | -0.13 |
| ND+Cr^3+^-1 | -8.50 | ND+Cr^3+^-4 | -8.20 |
| ND+Cr^3+^-2 | -8.29 | ND+Cr^3+^-5 | -7.74 |
| ND+Cr^3+^-3 | -7.52 | ND+Cr^3+^-6 | -7.36 |

**Table S2. The adsorption energies of the possible geometric structures of NDH-Cu^2+^ complexes in water solution.**

| NDH-Cu^2+^ complexes | ΔE_i_ | NDH-Cu^2+^ complexes | ΔE_i_ |
| --- | --- | --- | --- |
| NDH-1+ Cu^2+^ | 0 | NDH-2+ Cu^2+^ | 0 |
| NDH+Cu^2+^-1 | -5.04 | NDH+Cu^2+^-4 | -4.95 |
| NDH+Cu^2+^-2 | -4.56 | NDH+Cu^2+^-5 | -4.68 |
| NDH+Cu^2+^-3 | -4.03 | NDH+Cu^2+^-6 | -4.45 |
